# Supplementary material for: Socioeconomic and health impacts of fall armyworm in Ethiopia
Source: PLoS One. 2021 Nov 4;16(11):e0257736. doi: 10.1371/journal.pone.0257736 (PMC8568106; doi:10.1371/journal.pone.0257736)
Supplement: S1 File — (PDF) [file pone.0257736.s001.pdf]

```

/*
Do file for the paper:
Zewdu Abro, Emily Kimathi, Hugo De Groote, Tadele Tefera, Subramanian Sevgan, Saliou Niassy, and Menale Kassie (2020). The socioeconomic and health impacts of Fall Armyworm in Ethiopia
Community survey data are submitted together with the paper
the datasets from the Central Statistical Agency of Ethiopia could be requested at
https://www.statsethiopia.gov.et/
*/

****************************************************************
**********Step 1: Analysing the community survey data**********
****************************************************************

local graphfont "Arial"
graph set eps fontface `graphfont'
graph set eps fontfaceserif `graphfont'
graph set eps  /*echo back preferences*/
graph set window fontface `graphfont'
graph set window fontfaceserif `graphfont'
graph set window /*echo back preferences*/


cd "Final_Dataset_FAW_Community_Survey"
//GPS information
use "Module_0_Kebele_Info_Consent_vr1.dta", clear
tempfile GPS_Info
save "`GPS_Info'"
import excel "GPS_information__FG_MME.xlsx", sheet("Z_points_wz_mme_summary") firstrow clear
rename NAME mme
label var mme "Maize Mega Environment"

tempfile mme_data
save "`mme_data'"


import excel "GPS_points_AEZ.xlsx", sheet("GPS_points_AEZ") firstrow clear
label define maize_mega 1   "Wet Upper Mid-altitude" ///
2   "Wet Lower Mid-altitude" ///
3   "Dry Mid-altitude" ///
4   "Wet Lowland" ///
```

```
5   "Dry Lowland" ///
6   "Highland"
label values maize_mega maize_mega

merge 1:1 region zone woreda kebele using "`mme_data'", keepusing(mme)
drop _merge
encode mme, gen(mme_new)
replace maize_mega=1 if FID==131 & maize_mega==0
replace maize_mega=6 if FID==13 & maize_mega==0
replace maize_mega=3 if FID==34 & maize_mega==0

drop mme*
label var maize_mega "Maize mega environment"

tempfile mme_data
save "`mme_data'"

//Characterstics of the participants

use "Module_0_Q2_Characterstics_FGD_Participants_vr1.dta", clear
tab m0_q2e //gender is not important here

//Maize prices

use "Module_7_Maize_Prices_vr1.dta", clear
sort region zone woreda kebele
rename serial_num27 serial_num

tempfile prices
save "`prices'"

//Stages of FAW attack

use "Module_4_q4_Stages_FAW_Attack_vr1.dta", clear

tab m4_q4

tempfile stage_FAW_Attack
save "`stage_FAW_Attack'"

//FAW control techniques
use "Module_2_FAW_q6_Control_Methods_vr1.dta", clear
gen control_method=m2_q6a

label define control_method 1 "Chemicals" 2 "Cultural methods" 3
```

```
"Mechanical control" 4 "Host resistance" 5 "Biological control" 6 "Biopesticides" 7 "Botanical extracts" 8 "Cropping sytems" 9 "No approach used"
label values control_method control_method
drop if m2_q6c==.
drop if control_method==9

merge m:1 region zone woreda kebele using "`mme_data'", keepusing(maize_mega)
drop if _merge==2
drop _merge

//Figure 3. Effectiveness of FAW control methods

table control_method maize_mega if control_method!=6 & control_method!=4, c(mean m2_q6c sd m2_q6c)

graph bar (mean) m2_q6c if control_method!=6 & control_method!=4, over(control_method, sort(m2_q6c) descending label(angle(forty_five) labsize(small))) bar(1, fcolor(%50) lcolor(%50)) blabel(bar, size(vsmall) format(%9.2f) fcolor()) ytitle(Score: 0=least and 10=most effective) ytitle(, size(small)) ylabel(, labsize(small)) title(, size(small)) subtitle(Panel A: Average, size(small)) legend(order(1 "2017/18" 2 "2018/19" 3 "2019/20") rows(1) size(small)) graphregion(fcolor(white) ifcolor(white) ilcolor(white)) plotregion(fcolor(white) lcolor(white) ifcolor(white)) name(Eff_Ethiopia, replace)

graph bar (mean) m2_q6c if control_method!=6 & control_method!=4 & maize_mega==1, over(control_method, sort(m2_q6c) descending label(angle(forty_five) labsize(small))) bar(1, fcolor(%50) lcolor(%50)) blabel(bar, size(vsmall) format(%9.2f) fcolor()) ytitle(Score: 0=least and 10=most effective) ytitle(, size(small)) ylabel(, labsize(small)) title(, size(small)) subtitle(Panel B: Wet Upper Mid-altitude, size(small)) legend(order(1 "2017/18" 2 "2018/19" 3 "2019/20") rows(1) size(small)) graphregion(fcolor(white) ifcolor(white) ilcolor(white)) plotregion(fcolor(white) lcolor(white) ifcolor(white)) name(WUMA, replace)


graph bar (mean) m2_q6c if control_method!=6 & control_method!=4 & maize_mega==2, over(control_method, gap(*4) sort(m2_q6c) descending label(angle(forty_five) labsize(small))) bar(1, fcolor(%50) lcolor(%50)) blabel(bar, size(vsmall) format(%9.2f) fcolor()) ytitle(Score: 0=least and 10=most effective) ytitle(, size(small)) ylabel(, labsize(small)) title(, size(small)) subtitle(Panel C: Wet Lower Mid-altitude,
```

```
size(small)) legend(order(1 "2017/18" 2 "2018/19" 3 "2019/20") rows(1) size(small)) graphregion(fcolor(white) ifcolor(white) ilcolor(white)) plotregion(fcolor(white) lcolor(white) ifcolor(white)) name(WLMA, replace)

graph bar (mean) m2_q6c if control_method!=6 & control_method!=4 & maize_mega==3, over(control_method, sort(m2_q6c) descending label(angle(forty_five) labsize(small))) bar(1, fcolor(%50) lcolor(%50)) blabel(bar, size(vsmall) format(%9.2f) fcolor()) ytitle(Score: 0=least and 10=most effective) ytitle(, size(small)) ylabel(, labsize(small)) title(, size(small)) subtitle(Panel D: Dry Mid-altitude, size(small)) legend(order(1 "2017/18" 2 "2018/19" 3 "2019/20") rows(1) size(small)) graphregion(fcolor(white) ifcolor(white) ilcolor(white)) plotregion(fcolor(white) lcolor(white) ifcolor(white)) name(DMA, replace)


graph bar (mean) m2_q6c if control_method!=6 & control_method!=4 & maize_mega==5, over(control_method, gap(*8.5) sort(m2_q6c) descending label(angle(forty_five) labsize(small))) bar(1, fcolor(%50) lcolor(%50)) blabel(bar, size(vsmall) format(%9.2f) fcolor()) ytitle(Score: 0=least and 10=most effective) ytitle(, size(small)) ylabel(, labsize(small)) title(, size(small)) subtitle(Panel E: Dry Lowland, size(small)) legend(order(1 "2017/18" 2 "2018/19" 3 "2019/20") rows(1) size(small)) graphregion(fcolor(white) ifcolor(white) ilcolor(white)) plotregion(fcolor(white) lcolor(white) ifcolor(white)) name(WL, replace)


graph bar (mean) m2_q6c if control_method!=6 & control_method!=4 & maize_mega==6, over(control_method, sort(m2_q6c) descending label(angle(forty_five) labsize(small))) bar(1, fcolor(%50) lcolor(%50)) blabel(bar, size(vsmall) format(%9.2f) fcolor()) ytitle(Score: 0=least and 10=most effective) ytitle(, size(small)) ylabel(, labsize(small)) title(, size(small)) subtitle(Panel F: Highland, size(small)) legend(order(1 "2017/18" 2 "2018/19" 3 "2019/20") rows(1) size(small)) graphregion(fcolor(white) ifcolor(white) ilcolor(white)) plotregion(fcolor(white) lcolor(white) ifcolor(white)) name(Highland, replace)


graph combine Eff_Ethiopia WUMA WLMA DMA WL Highland , ycommon xcommon


tempfile FAW_control_techniques
save "`FAW_control_techniques'"

```

```
use "Module_0_Q1_Number_FGD_Participants_vr1.dta", clear

tempfile Num_of_participants
save "`Num_of_participants'"


use "Module_2_FAW_q1_q4_Awareness_Knowledge_vr1.dta", clear
merge 1:1 region zone woreda kebele using "`Num_of_participants'", keepusing(m0_q1)
drop _merge
gen aware_FAW=m2_q1/m0_q1*100
gen corr_identified_FAW=m2_q2/m0_q1*100
label var aware_FAW "Number of FGD participants who are aware of FAW (%)"
label var corr_identified_FAW "Number of FGD participants who correctly identified FAW (%)"
table region, c(mean aware_FAW mean corr_identified_FAW)

robvar aware_FAW, by(region) //unequal variance
robvar corr_identified_FAW, by(region) //unequal variance

oneway aware_FAW region, tabulate //unequal variance
anova aware_FAW i.region
anova corr_identified_FAW i.region

merge m:1 region zone woreda kebele using "`mme_data'", keepusing(maize_mega)
drop _merge

table maize_mega, c( mean aware_FAW mean corr_identified_FAW)


robvar aware_FAW, by(maize_mega) //unequal variance
robvar corr_identified_FAW, by(maize_mega) //unequal variance
oneway aware_FAW maize_mega, tabulate //unequal variance
anova aware_FAW i.maize_mega
anova corr_identified_FAW i.maize_mega

//Table 3: Percentage of FGD participants who are aware and correctly identified FAW

table maize_mega, c(mean aware_FAW mean corr_identified_FAW)

tempfile FAW_Awareness_Know
save "`FAW_Awareness_Know'"
```

```
//Loss

use "Module_2_FAW_q5_Incidence_Season_vr1.dta", clear

sort region zone woreda kebele serial_num
replace serial_num = 2 in 50
replace serial_num = 3 in 51
replace serial_num = 4 in 52
replace serial_num = 5 in 53
replace serial_num = 6 in 54
replace serial_num = 1 in 175
replace serial_num = 2 in 176
replace serial_num = 3 in 177
replace serial_num = 4 in 178
replace serial_num = 5 in 179
replace serial_num = 6 in 180

replace m2_q5a="Meher season of 2017/18" if serial_num==5 & m2_q5a==""
replace m2_q5a="Belg season of 2017/18" if serial_num==6 & m2_q5a==""
replace m2_q5a="Meher season of 2018/19" if serial_num==3 & m2_q5a==""
replace m2_q5a="Belg season of 2018/19" if serial_num==4 & m2_q5a==""
replace m2_q5a="Meher season 2019/20" if serial_num==1 & m2_q5a==""
replace m2_q5a="Belg season of 2019/20" if serial_num==2 & m2_q5a==""


rename m2_q5a season

duplicates drop region zone woreda kebele serial_num season, force

merge m:1 region zone woreda kebele using "`mme_data'", keepusing(
maize_mega)
drop _merge

tab m2_q5e maize_mega if m2_q5e!=6, column nofreq  //Table 2

tempfile incidence_FAW
save "`incidence_FAW'"

use "Module_4_q1_Maize_Yield_Loss_vr1.dta", clear
sort region zone woreda kebele serial_num
replace serial_num21 = 1 in 786
replace serial_num21 = 1 in 804
replace m4_q1a="Meher season 2019/20" if serial_num==1 & m4_q1a==""

```

```
merge m:1 region zone woreda kebele using "`GPS_Info'" , keepusing (latitude longitude altitude)
drop _merge
merge m:1 region zone woreda kebele using "`mme_data'", keepusing(maize_mega)
drop _merge
merge m:1 region zone woreda kebele using "`Num_of_participants'", keepusing(m0_q1)
drop _merge
rename (serial_num21 m4_q1a) (serial_num season)
merge 1:1 region zone woreda kebele serial_num season using "`incidence_FAW'", keepusing(m2_q5b m2_q5c m2_q5d m2_q5e)
drop if _merge==2
drop _merge
merge 1:1 region zone woreda kebele serial_num using "`expert_loss_ass'", keepusing(m2_qe m2_qj diff_yield_exp)
drop if _merge==2
drop _merge

foreach v of var m4_q1e m2_q5c {
replace `v'=`v'/100
}

egen mean=mean(m4_q1e)
replace m4_q1e=mean if m4_q1e==. & m2_q5c<.
drop mean

egen m2_q5c1=mean(m2_q5c), by(region zone woreda season)
replace m2_q5c=m2_q5c1 if m2_q5c==. & m4_q1e!=.
drop m2_q5c1
egen m2_q5c1=mean(m2_q5c), by(region zone woreda)
replace m2_q5c=m2_q5c1 if m2_q5c==. & m4_q1e!=.
drop m2_q5c1

gen year=1 if season=="Meher season of 2017/18"
replace year=1 if season=="Belg season of 2017/18"
replace year=2 if season=="Meher season of 2018/19"
replace year=2 if season=="Belg season of 2018/19"
replace year=3 if season=="Meher season 2019/20"
replace year=3 if season=="Belg season of 2019/20"
label define year 1 "2017/18" 2 "2018/19" 3 "2019/20"
label values year year
label var year "Year of data points"
```

```
gen FGD_part_Affected=m2_q5d/m0_q1*100
label var FGD_part_Affected "Number of FGD participants affected by FAW (%)"

//Figure 4

d m2_q5c m4_q1e
sum m2_q5c m4_q1e
merge m:1 region zone woreda kebele serial_num using "`prices'", keepusing(m7_q1b)
drop _merge
replace m7_q1b=9 if m7_q1b==90

gen maize_price=m7_q1b
label var maize_price "Maize price (ETB/kg)"

order region zone woreda kebele enumerator supervisor latitude longitude altitude

gen q_prod_actual=m4_q1b*m4_q1c

label var q_prod_actual "Actual production of the average farmers (kg)"

gen q_prod_potential=m4_q1b*m4_q1d
label var q_prod_potential "Production of the average farmers with no production constraints (kg)"

gen q_prod_No_FAW=m4_q1b*(m4_q1c+m4_q1c*m4_q1e)
label var q_prod_No_FAW "Quantity of production had FAW did not affect farmers (kg)"
gen Maize_prod_loss=1-(q_prod_actual/q_prod_No_FAW)
label var Maize_prod_loss "Maize production loss due to FAW (%)"

gen yield_loss=m4_q1e*100
label var yield_loss "Yield loss reported by farmers (%)"

gen diff_yield= m4_q1d-m4_q1c
label var diff_yield "Differences in yield (expected-actual) (kg)"

gen farmers_affected=m2_q5c
egen farmers_affected1=mean(farmers_affected), by(region zone woreda)
replace farmers_affected=farmers_affected1 if farmers_affected==. & q_prod_No_FAW!=.
drop farmers_affected1
label var farmers_affected "Farmers affected by FAW (%)"
```

```
replace Maize_prod_loss=Maize_prod_loss*100
replace farmers_affected=farmers_affected*100

gen yield_loss_exp=m2_qe
label var yield_loss_exp "Yield loss reported by farmers (%) experts estimate"
gen farmers_affected_exp=m2_qj
label var farmers_affected_exp "Farmers affected by FAW (%) experts estimate"
gen loss_stemborer=m4_q1f
label var loss_stemborer "Losses due to stemborer (%)"

keep region zone woreda kebele latitude longitude altitude serial_num maize_mega season year yield_loss_exp farmers_affected_exp yield_loss FGD_part_Affected maize_price q_prod_actual q_prod_potential q_prod_No_FAW Maize_prod_loss farmers_affected m4_q1d m4_q1c diff_yield diff_yield_exp NDVI NDVI Temp Rain FAWsuitability loss_stemborer

gen zone_id=.

replace zone_id=309 if zone==1
replace zone_id=302 if zone==2
replace zone_id=304 if zone==3
replace zone_id=301 if zone==4
replace zone_id=307 if zone==5
replace zone_id=417 if zone==6
replace zone_id=404 if zone==7
replace zone_id=402 if zone==8
replace zone_id=412 if zone==9
replace zone_id=410 if zone==10
replace zone_id=409 if zone==11
replace zone_id=408 if zone==12
replace zone_id=407 if zone==13
replace zone_id=403 if zone==14
replace zone_id=405 if zone==15
replace zone_id=401 if zone==16
replace zone_id=701 if zone==17
replace zone_id=702 if zone==18
replace zone_id=703 if zone==19
replace zone_id=704 if zone==20
replace zone_id=706 if zone==21

label define zone_id 309 "Awi" 302  "South Gonder" 304  "South welo" 301 "North Gonder" 307 "West Gojam" 417     "West Arsi" 404 "Jimma" 402
```

```
 "East Wellega" 412  "Borena/Guji" 410 "East Hararge" 409 "West Hararge" 408 "Arsi" 407  "East Shoa" 403 "Illuababora" 405 "West Shoa" 401 "West Wellega" 701 "Gurage" 702 "Hadiya" 703  "KAT" 704 "Sidama" 706  "Wolayta"
label values zone_id zone_id

recode region (1=3)(2=4) (3=7), gen(REG)
label define REG 3 "Amhara" 4 "Oromia" 7 "SNNP"
label values REG REG

anova farmers_affected maize_mega##year //MME significant: 5%
anova yield_loss maize_mega##year //MME significant: 1%

table year if woreda==35, c(mean farmers_affected mean yield_loss)
table maize_mega, c(mean m4_q1d mean m4_q1c mean diff_yield) //yield gap
sum m4_q1d m4_q1c diff_yield


gen yeild_loss_FAW=(diff_yield)*(yield_loss/100)
table maize_mega, c(mean yeild_loss_FAW)
replace yeild_loss_FAW=yeild_loss_FAW/1000
label var yeild_loss_FAW "Yield loss due to FAW (tonnes/ha)"
drop yeild_loss_FAW

//Table 5

table maize_mega year, c(mean farmers_affected semean farmers_affected) col row

//Table 6

table maize_mega year, c(mean yield_loss semean yield_loss)  col row
table maize_mega year, c(mean diff_yield semean diff_yield) col row
table maize_mega year, c(mean farmers_affected_exp semean farmers_affected_exp) col row
table maize_mega year, c(mean yield_loss_exp semean yield_loss_exp) col row
table maize_mega year, c(mean diff_yield_exp semean diff_yield_exp) col row
table maize_mega, c(mean m4_q1d mean m4_q1c)
table maize_mega, c(semean m4_q1d semean m4_q1c) col row
table maize_mega, c(mean diff_yield semean diff_yield) col row

collapse (mean) yield_loss_exp farmers_affected_exp farmers_affected
```

```
yield_loss diff_yield diff_yield_exp maize_price, by (maize_mega year)

label var yield_loss "Yield loss reported by farmers (%)"
label var farmers_affected "Farmers affected (%)"
label var maize_price "Maize price (ETB/kg)"
label var diff_yield "Differences in yield (expected-actual) (kg)"
label var diff_yield_exp "Differences in yield (expected-actual) (kg)
experts"
label var farmers_affected_exp "Farmers affected by FAW (%) experts
estimate"
label var yield_loss_exp "Yield loss reported by farmers (%) experts
estimate"

tempfile Loss_farmers_affected
save "`Loss_farmers_affected'"


************************************************************************
******
**********Step 2: link the community survey data with CSA
datasetws**********
************************************************************************
******

//link the survey data with the CSA data: 2017/18, 2018/19, and 2019/20


import excel "New_Codes_2011_EC_Onwards_Code_books.xlsx", sheet(
"new_mapping") firstrow clear

keep woreda_id1 woreda_id
destring woreda_id1, replace
destring woreda_id, replace

mkmat woreda_id1 woreda_id, matrix(cf1)     /*new mapping: <=2010 to
>=2011*/
global cfsz1 = rowsof(cf1)

import excel "New_Codes_2011_EC_Onwards_Code_books.xlsx", sheet(
"zone_2010to2011_mapping") firstrow clear
drop old_zone_name
destring new_zone_code, replace

mkmat old_zone_code new_zone_code, matrix(cf2)      /*new mapping:
<=2010 to >=2011*/
```

```
global cfsz2 = rowsof(cf2)


import excel "Woreda_mapping_to_maize_mega_environment_vr4.xlsx",
sheet("agroecologies") firstrow clear
//keep woreda_id maize_mega
keep woreda_id maize_mega ADM3_PCODE_new Latitude Longitude
order woreda_id maize_mega ADM3_PCODE_new Latitude Longitude
mkmat woreda_id maize_mega ADM3_PCODE_new Latitude Longitude, matrix(
cf3)   /*new mapping: agroecologies*/
global cfsz3 = rowsof(cf3)


//import spss using "HOUSEHOLD INFORMATION_MEHER 2012 EC.sav", clear

import spss using "HOUSEHOLD INFORMATION_MEHER 2012 EC.sav", clear

duplicates drop REG ZONE DIST FA EA HH, force

tempfile HHSIZE
save "`HHSIZE'"

//import spss using "FIELD INFORMATION_MEHER 2012 EC.sav", clear

import spss using "FIELD INFORMATION_MEHER 2012 EC.sav", clear

duplicates drop

merge m:1 REG ZONE DIST FA EA HH using "`HHSIZE'", keepusing(HHSIZE)
drop if _merge==2
drop _merge

keep if CROP==2
keep REG ZONE DIST FA EA HH HHSIZE FWEIGHT AREAH PRODC
replace PRODC=PRODC/1000
label var PRODC "Production (kg)"
gen year=3
table year [pw=FWEIGHT], c(sum PRODC ) format (%20.0g)

gen zero=0
egen zone_id=concat(zero REG zero ZONE)
destring zone_id, replace
replace zone_id=310 if zone_id==3010
replace zone_id=311 if zone_id==3011
replace zone_id=312 if zone_id==3012
```

```
replace zone_id=313 if zone_id==3013
replace zone_id=314 if zone_id==3014
replace zone_id=315 if zone_id==3015
replace zone_id=316 if zone_id==3016
replace zone_id=410 if zone_id==4010

replace zone_id=411 if zone_id==4011
replace zone_id=412 if zone_id==4012
replace zone_id=413 if zone_id==4013
replace zone_id=414 if zone_id==4014
replace zone_id=415 if zone_id==4015
replace zone_id=416 if zone_id==4016
replace zone_id=417 if zone_id==4017
replace zone_id=418 if zone_id==4018
replace zone_id=419 if zone_id==4019
replace zone_id=420 if zone_id==4020

replace zone_id=511 if zone_id==5011

replace zone_id=710 if zone_id==7010
replace zone_id=711 if zone_id==7011
replace zone_id=712 if zone_id==7012
replace zone_id=713 if zone_id==7013
replace zone_id=714 if zone_id==7014
replace zone_id=715 if zone_id==7015
replace zone_id=716 if zone_id==7016
replace zone_id=717 if zone_id==7017
replace zone_id=718 if zone_id==7018
replace zone_id=719 if zone_id==7019
replace zone_id=720 if zone_id==7020


label values zone_id zone_id

//gen newvar1 = real(substr(string(zone_id),1,3,4)) if ZONE>9

egen woreda_id=concat(zone_id zero DIST)

//egen woreda_id=concat(zero REG zero ZONE zero DIST)
destring woreda_id, replace

label values woreda_id woreda_id
replace woreda_id=10210 if woreda_id==102010
replace woreda_id=30211 if woreda_id==302011
replace woreda_id=30214 if woreda_id==302014
```

```
replace woreda_id=30215 if woreda_id==302015
replace woreda_id=30217 if woreda_id==302017
replace woreda_id=30310 if woreda_id==303010
replace woreda_id=30311 if woreda_id==303011
replace woreda_id=30312 if woreda_id==303012
replace woreda_id=30410 if woreda_id==304010
replace woreda_id=30411 if woreda_id==304011
replace woreda_id=30412 if woreda_id==304012
replace woreda_id=30413 if woreda_id==304013
replace woreda_id=30414 if woreda_id==304014
replace woreda_id=30415 if woreda_id==304015
replace woreda_id=30416 if woreda_id==304016
replace woreda_id=30417 if woreda_id==304017
replace woreda_id=30419 if woreda_id==304019
replace woreda_id=30420 if woreda_id==304020
replace woreda_id=30424 if woreda_id==304024
replace woreda_id=30510 if woreda_id==305010
replace woreda_id=30512 if woreda_id==305012
replace woreda_id=30513 if woreda_id==305013
replace woreda_id=30514 if woreda_id==305014
replace woreda_id=30515 if woreda_id==305015
replace woreda_id=30517 if woreda_id==305017
replace woreda_id=30518 if woreda_id==305018
replace woreda_id=30519 if woreda_id==305019
replace woreda_id=30521 if woreda_id==305021
replace woreda_id=30610 if woreda_id==306010
replace woreda_id=30611 if woreda_id==306011
replace woreda_id=30612 if woreda_id==306012
replace woreda_id=30613 if woreda_id==306013
replace woreda_id=30614 if woreda_id==306014
replace woreda_id=30616 if woreda_id==306016
replace woreda_id=30617 if woreda_id==306017
replace woreda_id=30619 if woreda_id==306019
replace woreda_id=30710 if woreda_id==307010
replace woreda_id=30711 if woreda_id==307011
replace woreda_id=30712 if woreda_id==307012
replace woreda_id=30713 if woreda_id==307013
replace woreda_id=30716 if woreda_id==307016
replace woreda_id=30717 if woreda_id==307017
replace woreda_id=31510 if woreda_id==315010
replace woreda_id=31511 if woreda_id==315011
replace woreda_id=31512 if woreda_id==315012
replace woreda_id=31513 if woreda_id==315013
replace woreda_id=40110 if woreda_id==401010
replace woreda_id=40111 if woreda_id==401011
```

```
replace woreda_id=40114 if woreda_id==401014
replace woreda_id=40115 if woreda_id==401015
replace woreda_id=40116 if woreda_id==401016
replace woreda_id=40118 if woreda_id==401018
replace woreda_id=40210 if woreda_id==402010
replace woreda_id=40211 if woreda_id==402011
replace woreda_id=40212 if woreda_id==402012
replace woreda_id=40213 if woreda_id==402013
replace woreda_id=40214 if woreda_id==402014
replace woreda_id=40215 if woreda_id==402015
replace woreda_id=40216 if woreda_id==402016
replace woreda_id=40217 if woreda_id==402017
replace woreda_id=40310 if woreda_id==403010
replace woreda_id=40311 if woreda_id==403011
replace woreda_id=40312 if woreda_id==403012
replace woreda_id=40314 if woreda_id==403014
replace woreda_id=40410 if woreda_id==404010
replace woreda_id=40411 if woreda_id==404011
replace woreda_id=40412 if woreda_id==404012
replace woreda_id=40413 if woreda_id==404013
replace woreda_id=40414 if woreda_id==404014
replace woreda_id=40415 if woreda_id==404015
replace woreda_id=40416 if woreda_id==404016
replace woreda_id=40418 if woreda_id==404018
replace woreda_id=40419 if woreda_id==404019
replace woreda_id=40420 if woreda_id==404020
replace woreda_id=40421 if woreda_id==404021
replace woreda_id=40510 if woreda_id==405010
replace woreda_id=40511 if woreda_id==405011
replace woreda_id=40512 if woreda_id==405012
replace woreda_id=40513 if woreda_id==405013
replace woreda_id=40514 if woreda_id==405014
replace woreda_id=40515 if woreda_id==405015
replace woreda_id=40516 if woreda_id==405016
replace woreda_id=40517 if woreda_id==405017
replace woreda_id=40518 if woreda_id==405018
replace woreda_id=40520 if woreda_id==405020
replace woreda_id=40521 if woreda_id==405021
replace woreda_id=40522 if woreda_id==405022
replace woreda_id=40611 if woreda_id==406011
replace woreda_id=40710 if woreda_id==407010
replace woreda_id=40711 if woreda_id==407011
replace woreda_id=40810 if woreda_id==408010
replace woreda_id=40811 if woreda_id==408011
replace woreda_id=40812 if woreda_id==408012
```

```
replace woreda_id=40813 if woreda_id==408013
replace woreda_id=40814 if woreda_id==408014
replace woreda_id=40815 if woreda_id==408015
replace woreda_id=40816 if woreda_id==408016
replace woreda_id=40817 if woreda_id==408017
replace woreda_id=40819 if woreda_id==408019
replace woreda_id=40820 if woreda_id==408020
replace woreda_id=40821 if woreda_id==408021
replace woreda_id=40822 if woreda_id==408022
replace woreda_id=40823 if woreda_id==408023
replace woreda_id=40824 if woreda_id==408024
replace woreda_id=40825 if woreda_id==408025
replace woreda_id=40910 if woreda_id==409010
replace woreda_id=40911 if woreda_id==409011
replace woreda_id=40912 if woreda_id==409012
replace woreda_id=40913 if woreda_id==409013
replace woreda_id=40915 if woreda_id==409015
replace woreda_id=40916 if woreda_id==409016
replace woreda_id=40917 if woreda_id==409017
replace woreda_id=41010 if woreda_id==410010
replace woreda_id=41011 if woreda_id==410011
replace woreda_id=41012 if woreda_id==410012
replace woreda_id=41013 if woreda_id==410013
replace woreda_id=41014 if woreda_id==410014
replace woreda_id=41015 if woreda_id==410015
replace woreda_id=41016 if woreda_id==410016
replace woreda_id=41017 if woreda_id==410017
replace woreda_id=41018 if woreda_id==410018
replace woreda_id=41019 if woreda_id==410019
replace woreda_id=41111 if woreda_id==411011
replace woreda_id=41112 if woreda_id==411012
replace woreda_id=41113 if woreda_id==411013
replace woreda_id=41114 if woreda_id==411014
replace woreda_id=41115 if woreda_id==411015
replace woreda_id=41116 if woreda_id==411016
replace woreda_id=41117 if woreda_id==411017
replace woreda_id=41118 if woreda_id==411018
replace woreda_id=41210 if woreda_id==412010
replace woreda_id=41211 if woreda_id==412011
replace woreda_id=41212 if woreda_id==412012
replace woreda_id=41213 if woreda_id==412013
replace woreda_id=41310 if woreda_id==413010
replace woreda_id=41311 if woreda_id==413011
replace woreda_id=41410 if woreda_id==414010
replace woreda_id=41413 if woreda_id==414013
```

```
replace woreda_id=41415 if woreda_id==414015
replace woreda_id=41710 if woreda_id==417010
replace woreda_id=41711 if woreda_id==417011
replace woreda_id=41713 if woreda_id==417013
replace woreda_id=41715 if woreda_id==417015
replace woreda_id=41811 if woreda_id==418011
replace woreda_id=41812 if woreda_id==418012
replace woreda_id=41910 if woreda_id==419010
replace woreda_id=41911 if woreda_id==419011
replace woreda_id=41912 if woreda_id==419012
replace woreda_id=50211 if woreda_id==502011
replace woreda_id=50213 if woreda_id==502013
replace woreda_id=70110 if woreda_id==701010
replace woreda_id=70111 if woreda_id==701011
replace woreda_id=70112 if woreda_id==701012
replace woreda_id=70210 if woreda_id==702010
replace woreda_id=70410 if woreda_id==704010
replace woreda_id=70411 if woreda_id==704011
replace woreda_id=70412 if woreda_id==704012
replace woreda_id=70414 if woreda_id==704014
replace woreda_id=70415 if woreda_id==704015
replace woreda_id=70417 if woreda_id==704017
replace woreda_id=70418 if woreda_id==704018
replace woreda_id=70420 if woreda_id==704020
replace woreda_id=70610 if woreda_id==706010
replace woreda_id=70611 if woreda_id==706011
replace woreda_id=70612 if woreda_id==706012
replace woreda_id=70910 if woreda_id==709010
replace woreda_id=71010 if woreda_id==710010
replace woreda_id=71011 if woreda_id==710011
replace woreda_id=71012 if woreda_id==710012
replace woreda_id=71013 if woreda_id==710013
replace woreda_id=71014 if woreda_id==710014
replace woreda_id=71015 if woreda_id==710015
replace woreda_id=71110 if woreda_id==711010

tempfile maize_prod_2012ec
save "`maize_prod_2012ec'"

//import spss using "HOUSEHOLD INFORMATION_MEHER 2011 EC.sav", clear

import spss using "HOUSEHOLD INFORMATION_MEHER 2011 EC.sav", clear


duplicates drop REG ZONE DIST FA EA HH, force
```

```
tempfile HHSIZE
save "`HHSIZE'"

//import spss using "FIELD INFORMATION_MEHER 2011 EC.sav", clear

import spss using "FIELD INFORMATION_MEHER 2011 EC.sav", clear

duplicates drop

merge m:1 REG ZONE DIST FA EA HH using "`HHSIZE'", keepusing(HHSIZE)
drop if _merge==2
drop _merge

keep if CROP==2
keep REG ZONE DIST FA EA HH HHSIZE FWEIGHT AREAH PRODC
replace PRODC=PRODC/1000
label var PRODC "Production (kg)"
gen year=2

gen zero=0
egen zone_id=concat(zero REG zero ZONE)
destring zone_id, replace
replace zone_id=310 if zone_id==3010
replace zone_id=311 if zone_id==3011
replace zone_id=312 if zone_id==3012
replace zone_id=313 if zone_id==3013
replace zone_id=314 if zone_id==3014
replace zone_id=315 if zone_id==3015
replace zone_id=316 if zone_id==3016
replace zone_id=410 if zone_id==4010

replace zone_id=411 if zone_id==4011
replace zone_id=412 if zone_id==4012
replace zone_id=413 if zone_id==4013
replace zone_id=414 if zone_id==4014
replace zone_id=415 if zone_id==4015
replace zone_id=416 if zone_id==4016
replace zone_id=417 if zone_id==4017
replace zone_id=418 if zone_id==4018
replace zone_id=419 if zone_id==4019
replace zone_id=420 if zone_id==4020

replace zone_id=511 if zone_id==5011

```

```
replace zone_id=710 if zone_id==7010
replace zone_id=711 if zone_id==7011
replace zone_id=712 if zone_id==7012
replace zone_id=713 if zone_id==7013
replace zone_id=714 if zone_id==7014
replace zone_id=715 if zone_id==7015
replace zone_id=716 if zone_id==7016
replace zone_id=717 if zone_id==7017
replace zone_id=718 if zone_id==7018
replace zone_id=719 if zone_id==7019
replace zone_id=720 if zone_id==7020


label values zone_id zone_id

//gen newvar1 = real(substr(string(zone_id),1,3,4)) if ZONE>9

egen woreda_id=concat(zone_id zero DIST)

//egen woreda_id=concat(zero REG zero ZONE zero DIST)
destring woreda_id, replace


replace woreda_id=10210 if woreda_id==102010
replace woreda_id=30211 if woreda_id==302011
replace woreda_id=30214 if woreda_id==302014
replace woreda_id=30215 if woreda_id==302015
replace woreda_id=30217 if woreda_id==302017
replace woreda_id=30310 if woreda_id==303010
replace woreda_id=30311 if woreda_id==303011
replace woreda_id=30312 if woreda_id==303012
replace woreda_id=30410 if woreda_id==304010
replace woreda_id=30411 if woreda_id==304011
replace woreda_id=30412 if woreda_id==304012
replace woreda_id=30413 if woreda_id==304013
replace woreda_id=30414 if woreda_id==304014
replace woreda_id=30415 if woreda_id==304015
replace woreda_id=30416 if woreda_id==304016
replace woreda_id=30417 if woreda_id==304017
replace woreda_id=30419 if woreda_id==304019
replace woreda_id=30420 if woreda_id==304020
replace woreda_id=30424 if woreda_id==304024
replace woreda_id=30510 if woreda_id==305010
replace woreda_id=30512 if woreda_id==305012
replace woreda_id=30513 if woreda_id==305013
```

```
replace woreda_id=30514 if woreda_id==305014
replace woreda_id=30515 if woreda_id==305015
replace woreda_id=30517 if woreda_id==305017
replace woreda_id=30518 if woreda_id==305018
replace woreda_id=30519 if woreda_id==305019
replace woreda_id=30521 if woreda_id==305021
replace woreda_id=30610 if woreda_id==306010
replace woreda_id=30611 if woreda_id==306011
replace woreda_id=30612 if woreda_id==306012
replace woreda_id=30613 if woreda_id==306013
replace woreda_id=30614 if woreda_id==306014
replace woreda_id=30616 if woreda_id==306016
replace woreda_id=30617 if woreda_id==306017
replace woreda_id=30619 if woreda_id==306019
replace woreda_id=30710 if woreda_id==307010
replace woreda_id=30711 if woreda_id==307011
replace woreda_id=30712 if woreda_id==307012
replace woreda_id=30713 if woreda_id==307013
replace woreda_id=30716 if woreda_id==307016
replace woreda_id=30717 if woreda_id==307017
replace woreda_id=31510 if woreda_id==315010
replace woreda_id=31511 if woreda_id==315011
replace woreda_id=31512 if woreda_id==315012
replace woreda_id=31513 if woreda_id==315013
replace woreda_id=40110 if woreda_id==401010
replace woreda_id=40111 if woreda_id==401011
replace woreda_id=40114 if woreda_id==401014
replace woreda_id=40115 if woreda_id==401015
replace woreda_id=40116 if woreda_id==401016
replace woreda_id=40118 if woreda_id==401018
replace woreda_id=40210 if woreda_id==402010
replace woreda_id=40211 if woreda_id==402011
replace woreda_id=40212 if woreda_id==402012
replace woreda_id=40213 if woreda_id==402013
replace woreda_id=40214 if woreda_id==402014
replace woreda_id=40215 if woreda_id==402015
replace woreda_id=40216 if woreda_id==402016
replace woreda_id=40217 if woreda_id==402017
replace woreda_id=40310 if woreda_id==403010
replace woreda_id=40311 if woreda_id==403011
replace woreda_id=40312 if woreda_id==403012
replace woreda_id=40314 if woreda_id==403014
replace woreda_id=40410 if woreda_id==404010
replace woreda_id=40411 if woreda_id==404011
replace woreda_id=40412 if woreda_id==404012
```

```
replace woreda_id=40413 if woreda_id==404013
replace woreda_id=40414 if woreda_id==404014
replace woreda_id=40415 if woreda_id==404015
replace woreda_id=40416 if woreda_id==404016
replace woreda_id=40418 if woreda_id==404018
replace woreda_id=40419 if woreda_id==404019
replace woreda_id=40420 if woreda_id==404020
replace woreda_id=40421 if woreda_id==404021
replace woreda_id=40510 if woreda_id==405010
replace woreda_id=40511 if woreda_id==405011
replace woreda_id=40512 if woreda_id==405012
replace woreda_id=40513 if woreda_id==405013
replace woreda_id=40514 if woreda_id==405014
replace woreda_id=40515 if woreda_id==405015
replace woreda_id=40516 if woreda_id==405016
replace woreda_id=40517 if woreda_id==405017
replace woreda_id=40518 if woreda_id==405018
replace woreda_id=40520 if woreda_id==405020
replace woreda_id=40521 if woreda_id==405021
replace woreda_id=40522 if woreda_id==405022
replace woreda_id=40611 if woreda_id==406011
replace woreda_id=40710 if woreda_id==407010
replace woreda_id=40711 if woreda_id==407011
replace woreda_id=40810 if woreda_id==408010
replace woreda_id=40811 if woreda_id==408011
replace woreda_id=40812 if woreda_id==408012
replace woreda_id=40813 if woreda_id==408013
replace woreda_id=40814 if woreda_id==408014
replace woreda_id=40815 if woreda_id==408015
replace woreda_id=40816 if woreda_id==408016
replace woreda_id=40817 if woreda_id==408017
replace woreda_id=40819 if woreda_id==408019
replace woreda_id=40820 if woreda_id==408020
replace woreda_id=40821 if woreda_id==408021
replace woreda_id=40822 if woreda_id==408022
replace woreda_id=40823 if woreda_id==408023
replace woreda_id=40824 if woreda_id==408024
replace woreda_id=40825 if woreda_id==408025
replace woreda_id=40910 if woreda_id==409010
replace woreda_id=40911 if woreda_id==409011
replace woreda_id=40912 if woreda_id==409012
replace woreda_id=40913 if woreda_id==409013
replace woreda_id=40915 if woreda_id==409015
replace woreda_id=40916 if woreda_id==409016
replace woreda_id=40917 if woreda_id==409017
```

```
replace woreda_id=41010 if woreda_id==410010
replace woreda_id=41011 if woreda_id==410011
replace woreda_id=41012 if woreda_id==410012
replace woreda_id=41013 if woreda_id==410013
replace woreda_id=41014 if woreda_id==410014
replace woreda_id=41015 if woreda_id==410015
replace woreda_id=41016 if woreda_id==410016
replace woreda_id=41017 if woreda_id==410017
replace woreda_id=41018 if woreda_id==410018
replace woreda_id=41019 if woreda_id==410019
replace woreda_id=41111 if woreda_id==411011
replace woreda_id=41112 if woreda_id==411012
replace woreda_id=41113 if woreda_id==411013
replace woreda_id=41114 if woreda_id==411014
replace woreda_id=41115 if woreda_id==411015
replace woreda_id=41116 if woreda_id==411016
replace woreda_id=41117 if woreda_id==411017
replace woreda_id=41118 if woreda_id==411018
replace woreda_id=41210 if woreda_id==412010
replace woreda_id=41211 if woreda_id==412011
replace woreda_id=41212 if woreda_id==412012
replace woreda_id=41213 if woreda_id==412013
replace woreda_id=41310 if woreda_id==413010
replace woreda_id=41311 if woreda_id==413011
replace woreda_id=41410 if woreda_id==414010
replace woreda_id=41413 if woreda_id==414013
replace woreda_id=41415 if woreda_id==414015
replace woreda_id=41710 if woreda_id==417010
replace woreda_id=41711 if woreda_id==417011
replace woreda_id=41713 if woreda_id==417013
replace woreda_id=41715 if woreda_id==417015
replace woreda_id=41811 if woreda_id==418011
replace woreda_id=41812 if woreda_id==418012
replace woreda_id=41910 if woreda_id==419010
replace woreda_id=41911 if woreda_id==419011
replace woreda_id=41912 if woreda_id==419012
replace woreda_id=50211 if woreda_id==502011
replace woreda_id=50213 if woreda_id==502013
replace woreda_id=70110 if woreda_id==701010
replace woreda_id=70111 if woreda_id==701011
replace woreda_id=70112 if woreda_id==701012
replace woreda_id=70210 if woreda_id==702010
replace woreda_id=70410 if woreda_id==704010
replace woreda_id=70411 if woreda_id==704011
replace woreda_id=70412 if woreda_id==704012
```

```
replace woreda_id=70414 if woreda_id==704014
replace woreda_id=70415 if woreda_id==704015
replace woreda_id=70417 if woreda_id==704017
replace woreda_id=70418 if woreda_id==704018
replace woreda_id=70420 if woreda_id==704020
replace woreda_id=70610 if woreda_id==706010
replace woreda_id=70611 if woreda_id==706011
replace woreda_id=70612 if woreda_id==706012
replace woreda_id=70910 if woreda_id==709010
replace woreda_id=71010 if woreda_id==710010
replace woreda_id=71011 if woreda_id==710011
replace woreda_id=71012 if woreda_id==710012
replace woreda_id=71013 if woreda_id==710013
replace woreda_id=71014 if woreda_id==710014
replace woreda_id=71015 if woreda_id==710015
replace woreda_id=71110 if woreda_id==711010

replace woreda_id=40112 if woreda_id==401012
replace woreda_id=40113 if woreda_id==401013
replace woreda_id=40123 if woreda_id==401023
replace woreda_id=40519 if woreda_id==405019
replace woreda_id=40818 if woreda_id==408018
replace woreda_id=41416 if woreda_id==414016

label values woreda_id woreda_id

tempfile maize_prod_2011ec
save "`maize_prod_2011ec'"

//import spss using "HOUSEHOLD INFORMATION_MEHER 2010 EC.sav", clear

import spss using "HOUSEHOLD INFORMATION_MEHER 2010 EC.sav", clear


duplicates drop REG ZONE DIST FA EA HH, force

tempfile HHSIZE
save "`HHSIZE'"

//import spss using "FIELD INFORMATION_MEHER 2010 EC.sav", clear

import spss using "FIELD INFORMATION_MEHER 2010 EC.sav", clear

duplicates drop

```

```
merge m:1 REG ZONE DIST FA EA HH using "`HHSIZE'", keepusing(HHSIZE)
drop if _merge==2
drop _merge

keep if CROP==2
keep REG ZONE DIST FA EA HH HHSIZE FWEIGHT AREAH PRODC
replace PRODC=PRODC/1000
label var PRODC "Production (kg)"
drop if PRODC==.
gen year=1

gen zero=0
egen zone_id1=concat(zero REG zero ZONE)
destring zone_id1, replace


replace zone_id1=310 if zone_id1==3010
replace zone_id1=312 if zone_id1==3012
replace zone_id1=410 if zone_id1==4010
replace zone_id1=411 if zone_id1==4011
replace zone_id1=412 if zone_id1==4012
replace zone_id1=413 if zone_id1==4013
replace zone_id1=414 if zone_id1==4014
replace zone_id1=417 if zone_id1==4017
replace zone_id1=418 if zone_id1==4018
replace zone_id1=419 if zone_id1==4019
replace zone_id1=710 if zone_id1==7010
replace zone_id1=711 if zone_id1==7011
replace zone_id1=712 if zone_id1==7012
replace zone_id1=717 if zone_id1==7017
replace zone_id1=718 if zone_id1==7018
replace zone_id1=719 if zone_id1==7019
replace zone_id1=720 if zone_id1==7020
replace zone_id1=721 if zone_id1==7021
replace zone_id1=725 if zone_id1==7025
label values zone_id1 zone_id1

egen woreda_id1=concat(zone_id1 zero DIST)
destring woreda_id1, replace

replace woreda_id1=10210 if woreda_id1==102010

replace woreda_id1=30110 if woreda_id1==301010
replace woreda_id1=30111 if woreda_id1==301011
replace woreda_id1=30112 if woreda_id1==301012
```

```
replace woreda_id1=30113 if woreda_id1==301013
replace woreda_id1=30114 if woreda_id1==301014
replace woreda_id1=30115 if woreda_id1==301015
replace woreda_id1=30116 if woreda_id1==301016
replace woreda_id1=30119 if woreda_id1==301019
replace woreda_id1=30120 if woreda_id1==301020
replace woreda_id1=30121 if woreda_id1==301021
replace woreda_id1=30211 if woreda_id1==302011
replace woreda_id1=30310 if woreda_id1==303010
replace woreda_id1=30311 if woreda_id1==303011
replace woreda_id1=30411 if woreda_id1==304011
replace woreda_id1=30412 if woreda_id1==304012
replace woreda_id1=30414 if woreda_id1==304014
replace woreda_id1=30416 if woreda_id1==304016
replace woreda_id1=30419 if woreda_id1==304019
replace woreda_id1=30510 if woreda_id1==305010
replace woreda_id1=30512 if woreda_id1==305012
replace woreda_id1=30513 if woreda_id1==305013
replace woreda_id1=30515 if woreda_id1==305015
replace woreda_id1=30518 if woreda_id1==305018
replace woreda_id1=30610 if woreda_id1==306010
replace woreda_id1=30611 if woreda_id1==306011
replace woreda_id1=30612 if woreda_id1==306012
replace woreda_id1=30613 if woreda_id1==306013
replace woreda_id1=30614 if woreda_id1==306014
replace woreda_id1=30616 if woreda_id1==306016
replace woreda_id1=30617 if woreda_id1==306017
replace woreda_id1=30710 if woreda_id1==307010
replace woreda_id1=30711 if woreda_id1==307011
replace woreda_id1=30712 if woreda_id1==307012
replace woreda_id1=30713 if woreda_id1==307013
replace woreda_id1=40110 if woreda_id1==401010
replace woreda_id1=40111 if woreda_id1==401011
replace woreda_id1=40112 if woreda_id1==401012
replace woreda_id1=40113 if woreda_id1==401013
replace woreda_id1=40114 if woreda_id1==401014
replace woreda_id1=40115 if woreda_id1==401015
replace woreda_id1=40116 if woreda_id1==401016
replace woreda_id1=40118 if woreda_id1==401018
replace woreda_id1=40119 if woreda_id1==401019
replace woreda_id1=40120 if woreda_id1==401020
replace woreda_id1=40210 if woreda_id1==402010
replace woreda_id1=40211 if woreda_id1==402011
replace woreda_id1=40212 if woreda_id1==402012
replace woreda_id1=40213 if woreda_id1==402013
```

```
replace woreda_id1=40214 if woreda_id1==402014
replace woreda_id1=40215 if woreda_id1==402015
replace woreda_id1=40216 if woreda_id1==402016
replace woreda_id1=40310 if woreda_id1==403010
replace woreda_id1=40311 if woreda_id1==403011
replace woreda_id1=40312 if woreda_id1==403012
replace woreda_id1=40313 if woreda_id1==403013
replace woreda_id1=40315 if woreda_id1==403015
replace woreda_id1=40316 if woreda_id1==403016
replace woreda_id1=40317 if woreda_id1==403017
replace woreda_id1=40318 if woreda_id1==403018
replace woreda_id1=40322 if woreda_id1==403022
replace woreda_id1=40323 if woreda_id1==403023
replace woreda_id1=40324 if woreda_id1==403024
replace woreda_id1=40410 if woreda_id1==404010
replace woreda_id1=40411 if woreda_id1==404011
replace woreda_id1=40412 if woreda_id1==404012
replace woreda_id1=40413 if woreda_id1==404013
replace woreda_id1=40414 if woreda_id1==404014
replace woreda_id1=40415 if woreda_id1==404015
replace woreda_id1=40416 if woreda_id1==404016
replace woreda_id1=40510 if woreda_id1==405010
replace woreda_id1=40511 if woreda_id1==405011
replace woreda_id1=40512 if woreda_id1==405012
replace woreda_id1=40513 if woreda_id1==405013
replace woreda_id1=40514 if woreda_id1==405014
replace woreda_id1=40516 if woreda_id1==405016
replace woreda_id1=40517 if woreda_id1==405017
replace woreda_id1=40519 if woreda_id1==405019
replace woreda_id1=40616 if woreda_id1==406016
replace woreda_id1=40710 if woreda_id1==407010
replace woreda_id1=40711 if woreda_id1==407011
replace woreda_id1=40712 if woreda_id1==407012
replace woreda_id1=40811 if woreda_id1==408011
replace woreda_id1=40812 if woreda_id1==408012
replace woreda_id1=40813 if woreda_id1==408013
replace woreda_id1=40814 if woreda_id1==408014
replace woreda_id1=40815 if woreda_id1==408015
replace woreda_id1=40816 if woreda_id1==408016
replace woreda_id1=40817 if woreda_id1==408017
replace woreda_id1=40818 if woreda_id1==408018
replace woreda_id1=40820 if woreda_id1==408020
replace woreda_id1=40821 if woreda_id1==408021
replace woreda_id1=40823 if woreda_id1==408023
replace woreda_id1=40910 if woreda_id1==409010
```

```
replace woreda_id1=40911 if woreda_id1==409011
replace woreda_id1=40912 if woreda_id1==409012
replace woreda_id1=40913 if woreda_id1==409013
replace woreda_id1=41010 if woreda_id1==410010
replace woreda_id1=41011 if woreda_id1==410011
replace woreda_id1=41012 if woreda_id1==410012
replace woreda_id1=41013 if woreda_id1==410013
replace woreda_id1=41014 if woreda_id1==410014
replace woreda_id1=41016 if woreda_id1==410016
replace woreda_id1=41110 if woreda_id1==411010
replace woreda_id1=41111 if woreda_id1==411011
replace woreda_id1=41112 if woreda_id1==411012
replace woreda_id1=41113 if woreda_id1==411013
replace woreda_id1=41114 if woreda_id1==411014
replace woreda_id1=41115 if woreda_id1==411015
replace woreda_id1=41116 if woreda_id1==411016
replace woreda_id1=41212 if woreda_id1==412012
replace woreda_id1=41213 if woreda_id1==412013
replace woreda_id1=41215 if woreda_id1==412015
replace woreda_id1=41216 if woreda_id1==412016
replace woreda_id1=41217 if woreda_id1==412017
replace woreda_id1=41310 if woreda_id1==413010
replace woreda_id1=41312 if woreda_id1==413012
replace woreda_id1=41313 if woreda_id1==413013
replace woreda_id1=41410 if woreda_id1==414010
replace woreda_id1=41710 if woreda_id1==417010
replace woreda_id1=41712 if woreda_id1==417012
replace woreda_id1=41810 if woreda_id1==418010
replace woreda_id1=41811 if woreda_id1==418011
replace woreda_id1=41910 if woreda_id1==419010
replace woreda_id1=70110 if woreda_id1==701010
replace woreda_id1=70111 if woreda_id1==701011
replace woreda_id1=70112 if woreda_id1==701012
replace woreda_id1=70113 if woreda_id1==701013
replace woreda_id1=70210 if woreda_id1==702010
replace woreda_id1=70410 if woreda_id1==704010
replace woreda_id1=70411 if woreda_id1==704011
replace woreda_id1=70414 if woreda_id1==704014
replace woreda_id1=70415 if woreda_id1==704015
replace woreda_id1=70417 if woreda_id1==704017
replace woreda_id1=70418 if woreda_id1==704018
replace woreda_id1=70420 if woreda_id1==704020
replace woreda_id1=70611 if woreda_id1==706011
replace woreda_id1=70612 if woreda_id1==706012
replace woreda_id1=70910 if woreda_id1==709010
```

```
replace woreda_id1=71010 if woreda_id1==710010
replace woreda_id1=71011 if woreda_id1==710011
replace woreda_id1=71012 if woreda_id1==710012
replace woreda_id1=71013 if woreda_id1==710013
replace woreda_id1=71014 if woreda_id1==710014
replace woreda_id1=71015 if woreda_id1==710015
replace woreda_id1=71110 if woreda_id1==711010
replace woreda_id1=72513 if woreda_id1==725013
replace woreda_id1=72514 if woreda_id1==725014
replace woreda_id1=72515 if woreda_id1==725015
replace woreda_id1=72516 if woreda_id1==725016

gen zone_id=.

forvalues  r = 1/$cfsz2 {
    replace zone_id=cf2[`r',2] if zone_id1==cf2[`r',1]
}

gen woreda_id=.

forvalues  r = 1/$cfsz1 {
    replace woreda_id=cf1[`r',2] if woreda_id1==cf1[`r',1]
}


label define year 1 "2017/18" 2 "2018/19" 3 "2019/20"
label values year year

append using "`maize_prod_2011ec'"
append using "`maize_prod_2012ec'"

order REG ZONE DIST FA EA HH zone_id woreda_id year

preserve

collapse (sum) AREAH PRODC [pw=FWEIGHT], by(REG year)

reshape wide AREAH PRODC, i(REG) j(year)

export excel using "Production_Area_Ethiopia_2017_2020.xlsx", firstrow
(variables) replace

restore

keep if REG==3|REG==4|REG==7
```

```

gen maize_mega=.
gen ADM3_PCODE_new=.
gen Latitude=.
gen Longitude=.

forvalues  r = 1/$cfsz3 {
    replace maize_mega=cf3[`r',2] if woreda_id==cf3[`r',1]
    replace ADM3_PCODE_new=cf3[`r',3] if woreda_id==cf3[`r',1]
    replace Latitude=cf3[`r',4] if woreda_id==cf3[`r',1]
    replace Longitude=cf3[`r',5] if woreda_id==cf3[`r',1]
}

//ADM3_PCODE_new Latitude Longitude

label var maize_mega "Maize mega environment"
label var zone_id "Unique zone identifiers"
label var woreda_id "Unique woreda identifier"
label var year "Survey years"


replace PRODC=PRODC*0.001
label var PRODC "Maize production (tonnes)"
format %10.6f PRODC

gen yield=PRODC/AREAH
label var yield "Yield (tonnes/ha)"

egen count_hh= tag(REG ZONE DIST FA EA HH year)
replace count_hh =. if count_hh==0
gen landsize=AREAH


preserve
collapse (sum) AREAH PRODC [pw=FWEIGHT], by(maize_mega year)
reshape wide AREAH PRODC, i(maize_mega) j(year)
order maize_mega AREAH1 AREAH2 AREAH3
format %15.0f AREAH* PRODC*
restore

collapse (sum) count_hh AREAH PRODC (mean) landsize [pw=FWEIGHT], by(
REG zone_id maize_mega year)

label define maize_mega ///
1   "Wet Upper Mid-altitude" ///
```

```
2   "Wet lower Mid-altitude" ///
3   "Dry Mid-altitude" ///
4   "Wet lowland" ///
5   "Dry Lowland" ///
6   "Highland"
label values maize_mega maize_mega

table maize_mega year, c(sum count_hh) col row format (%12.2g)
table maize_mega year, c(semean count_hh) col row format (%12.2g)

table maize_mega, c(mean landsize semean landsize) col row

merge m:1 year maize_mega using "`Loss_farmers_affected'", keepusing( yield_loss_exp farmers_affected_exp yield_loss farmers_affected maize_price diff_yield diff_yield_exp)

drop _merge

foreach v of var yield_loss_exp farmers_affected_exp farmers_affected yield_loss maize_price diff_yield diff_yield_exp {
egen `v'_1=mean(`v'), by(maize_mega)
replace `v'=`v'_1 if `v'==.
drop `v'_1
}

foreach v of var yield_loss_exp farmers_affected_exp farmers_affected yield_loss maize_price diff_yield diff_yield_exp {
egen `v'_1=mean(`v')
replace `v'=`v'_1 if `v'==.
drop `v'_1
}
replace maize_price=maize_price/26.108 //https://nbebank.com/wp-content/uploads/pdf/annualbulletin/report-2018-2019.pdf
replace maize_price=maize_price*1000
replace diff_yield=diff_yield*0.001
gen prod_loss=landsize*(diff_yield*(yield_loss/100))*(count_hh*( farmers_affected/100))
label var prod_loss "Maize production loss (tonnes): FGDs"
gen v_prod_loss=prod_loss*maize_price
label var v_prod_loss "Maize production loss (US$):FGDs"

replace diff_yield_exp=diff_yield_exp*0.001
gen prod_loss_exp=landsize*(diff_yield_exp*(yield_loss_exp/100))*( count_hh*(farmers_affected_exp/100))
```

```
label var prod_loss_exp "Maize production loss (tonnes): Experts"
gen v_prod_loss_exp=prod_loss_exp*maize_price
label var v_prod_loss_exp "Maize production loss (US$): Experts"

gen prod_loss_hec=prod_loss/AREAH
label var prod_loss_hec "Production loss (tonnes/ha)"
gen prod_loss_hec_exp=prod_loss_exp/AREAH
label var prod_loss_hec_exp "Production loss (tonnes/ha): Experts"

//Production loss
collapse (sum) prod_loss v_prod_loss (mean) prod_loss_hec, by(
maize_mega year)
reshape wide prod_loss v_prod_loss prod_loss_hec, i(maize_mega) j(year)
order maize_mega prod_loss1 prod_loss2 prod_loss3 v_prod_loss1
v_prod_loss2 v_prod_loss3
format %12.0g v_prod_loss1 v_prod_loss2 v_prod_loss3
```
