# Supplementary material for: Socioeconomic and health impacts of fall armyworm in Ethiopia
Source: PLoS One. 2021 Nov 4;16(11):e0257736. doi: 10.1371/journal.pone.0257736 (PMC8568106; doi:10.1371/journal.pone.0257736)
Supplement: S3 File — (DOCX) [file pone.0257736.s003.docx]

**S3. Appendix**

Table A1. Estimates of farmers affected and yield losses reported by agricultural experts (2017-2019)

| Maize mega-environments | Farmers affected (%) | Yield loss (%) |
| --- | --- | --- |
| Wet upper mid-altitudes | 55 | 32 |
| Wet lower mid-altitudes | 79 | 40 |
| Dry mid-altitudes | 44 | 32 |
| Dry lowlands | 67 | 43 |
| Highlands | 47 | 30 |
| Average | 51 | 32 |

Source: experts’ survey (2017-2019)

Table A2. Number of farmers producing maize

|  | Number of farmers (millions) | | |
| --- | --- | --- | --- |
| Agro-ecological zones | 2017 | 2018 | 2019 |
| Wet upper mid-altitudes | 3.28 | 3.33 | 3.62 |
| Wet lower mid-altitudes | 0.43 | 0.29 | 0.24 |
| Dry mid-altitudes | 1.24 | 1.11 | 1.51 |
| Wet lowlands | 0.10 | 0.16 | 0.13 |
| Dry lowlands | 0.41 | 0.30 | 0.29 |
| Highlands | 3.28 | 3.69 | 4.41 |
| Total | 8.75 | 8.87 | 10.20 |

Source: CSA’s agricultural sample survey.

Table A3. Average land size per household

| Agro-ecological zones | 2017 | 2018 | 2019 |
| --- | --- | --- | --- |
| Wet upper mid-altitudes | 0.11 | 0.13 | 0.12 |
| Wet lower mid-altitudes | 0.06 | 0.08 | 0.09 |
| Dry mid-altitudes | 0.14 | 0.14 | 0.15 |
| Wet lowlands | 0.09 | 0.21 | 0.21 |
| Dry lowlands | 0.11 | 0.11 | 0.08 |
| Highlands | 0.10 | 0.11 | 0.12 |
| Overall average | 0.11 | 0.12 | 0.12 |

Source: CSA’s agricultural sample survey.

Table A4. Zone and regional-level estimates of maize production losses

|  |  | Loss (tonnes) | | |
| --- | --- | --- | --- | --- |
| Region | Zone | 2017 | 2018 | 2019 |
| Amhara | Semen Gondar | 7,036 | 808 | 775 |
| Amhara | Debub Gondar | 5,698 | 8,267 | 12,989 |
| Amhara | Semen Wollo | 1,566 | 1,003 | 1,387 |
| Amhara | Debub Wollo | 1,024 | 2,963 | 2,827 |
| Amhara | Semen Shewa | 1,650 | 1,545 | 936 |
| Amhara | Misrak Gojjam | 8,777 | 9,244 | 11,778 |
| Amhara | Mirab Gojjam | 7,456 | 14,868 | 18,693 |
| Amhara | Waghimra | 1,835 | 715 | 485 |
| Amhara | Awi | 4,667 | 8,412 | 5,068 |
| Amhara | Oromia Liyu Zone | 1,764 | 235 | 504 |
| Amhara | Bahir Dar Liyu |  | 1,675 | 339 |
| Amhara | Argoba Liyu | 774 | 95 |  |
| Amhara | Dessie Town Administration |  | 481 | 60 |
| Amhara | Gondar Ketema Liyu Zone |  | 408 | 265 |
| Amhara | Maekelawi Gondar |  | 10,785 | 8,548 |
| Amhara | Mirab Gondar |  | 1,648 | 1,710 |
|  | Amhara | 42,245 | 63,150 | 66,364 |
| Oromia | Mirab Wollega | 6,652 | 5,470 | 5,360 |
| Oromia | Misrak Wollega | 6,338 | 7,544 | 5,964 |
| Oromia | Ilu Ababor | 10,688 | 3,721 | 4,947 |
| Oromia | Jimma | 4,278 | 11,470 | 11,230 |
| Oromia | Mirab Shewa | 3,784 | 4,555 | 6,356 |
| Oromia | Semen Shewa | 576 | 718 | 758 |
| Oromia | Misrak Shewa | 7,545 | 11,804 | 21,663 |
| Oromia | Arsi | 3,728 | 6,087 | 7,629 |
| Oromia | Mirab Hararghe | 1,751 | 4,356 | 3,369 |
| Oromia | Misrak Hararghe | 1,074 | 3,275 | 2,817 |
| Oromia | Bale | 4,613 | 4,044 | 4,652 |
| Oromia | Borena | 2,696 | 54 | 418 |
| Oromia | Debub Mirab Shewa | 3,522 | 2,421 | 3,251 |
| Oromia | Guji | 5,320 | 2,651 | 5,643 |
| Oromia | Mirab Guji |  | 1,130 | 1,713 |
| Oromia | Oromia Liyu Zone |  | 413 | 209 |
| Oromia | Mirab Arsi | 6,373 | 10,323 | 14,363 |
| Oromia | Kelem Wollega | 4,986 | 4,187 | 3,752 |
| Oromia | Horo Guduru Wollega | 9,859 | 8,064 | 4,531 |
| Oromia | Buno Bedele |  | 3,473 | 5,249 |
|  | Oromia | 83,783 | 95,759 | 113,874 |
| SNNP | Gurage | 3,072 | 4,281 | 5,074 |
| SNNP | Hadiya | 2,889 | 4,303 | 3,925 |
| SNNP | Kembata Tembaro | 1,356 | 1,350 | 954 |
| SNNP | Sidama | 1,248 | 2,211 | 5,844 |
| SNNP | Gedeo | 878 | 417 | 1,667 |
| SNNP | Welayta | 1,857 | 2,052 | 5,229 |
| SNNP | Debub Omo | 5,535 | 5,562 | 7,389 |
| SNNP | Sheka | 159 | 302 | 1,170 |
| SNNP | Kefa | 3,079 | 5,709 | 6,868 |
| SNNP | Gamo Gofa | 5,093 | 9,758 | 11,145 |
| SNNP | Bench Maji | 4,550 | 5,592 | 3,484 |
| SNNP | Yem Liyu | 2,027 | 1,279 | 622 |
| SNNP | Segen Akababi Hizboch | 9,058 | 6,883 | 5,572 |
| SNNP | Alaba Special | 5,235 | 3,999 | 3,184 |
| SNNP | Dawro | 1,203 | 954 | 1,864 |
| SNNP | Basketo Special | 2,728 | 623 | 302 |
| SNNP | Konta Liyu | 640 | 243 | 265 |
| SNNP | Silite | 5,565 | 7,987 | 8,857 |
|  | SNNP | 56,171 | 63,505 | 73,414 |
|  | Ethiopia **^¥^** | 182,199 | 222,414 | 253,652 |

**^¥^** Note that the grand total is slightly different from the total loss reported in Table 7. This is due to differences in the calculation of averages by maize mega-environments and zones.

Sources: authors’ estimate based on community survey and CSA’s agricultural sample survey.
