# Supplementary material for: Socioeconomic and health impacts of fall armyworm in Ethiopia
Source: PLoS One. 2021 Nov 4;16(11):e0257736. doi: 10.1371/journal.pone.0257736 (PMC8568106; doi:10.1371/journal.pone.0257736)
Supplement: Attachment — Submitted filename: PONE-D-21-14461_reviewer M1.1.pdf [file pone.0257736.s004.pdf]

PLOS ONE

The socioeconomic and health impacts of Fall Armyworm in Ethiopia

--Manuscript Draft--

**Manuscript Number:**	PONE-D-21-14461
**Article Type:**	Research Article
**Full Title:**	The socioeconomic and health impacts of Fall Armyworm in Ethiopia
**Short Title:**	Socioeconomic and health impacts of Fall Armyworm
**Corresponding Author:**	Menale Kassie<br>ICIPE: International Centre for Insect Physiology and Ecology<br>Nairobi, KENYA
**Keywords:**	Fall armyworm; maize production loss; food security; Health; community and household surveys; insecticides
**Abstract:**	Since 2016, fall armyworm (FAW) has threatened the fragile food systems and economic performance of sub-Saharan Africa. Yet, there is limited evidence on this transboundary pest's economic and food security impacts in the region. Additionally, the health and environmental consequences of the insecticides used to control FAW have not been studied. In this paper, we present evidence on the impacts of FAW on maize production, food security, and human and environmental health. We use a combination of an agroecology-based community survey and nationally representative data from an agricultural household survey to achieve our objectives. The results indicate that the pest causes an average annual loss of 36% in maize production, a reduction of 0.67 million tonnes of maize (0.225 million tonnes per year) between 2017 and 2019. The total economic loss is US $ 200 million, or 0.08% of the gross domestic product. The lost production could have met the per capita maize consumption of 4 million people. We also find that the application of insecticides to control FAW has more significant toxic effects on the environment than on humans. This paper highlights governments and development partners need to invest in more diverse FAW control strategies that reduce production losses while protecting the environment and biodiversity.
**Order of Authors:**	Zewdu Abro
	Emily Kimathi
	Hugo De Groote
	Tadele Tefera
	Subramanian Sevgan
	Saliou Niassy
	Menale Kassie
**Additional Information:**	
**Question**	**Response**
**Financial Disclosure**<br><br>Enter a financial disclosure statement that describes the sources of funding for the work included in this submission. Review the submission guidelines for detailed requirements. View published research articles from *PLOS ONE* for specific examples.<br><br>This statement is required for submission	This study was supported by the USAID Feed the Future IPM Innovation Lab, Virginia Tech (Grant No. AID-OAA-L-15-00001); the Norwegian Agency for Development Cooperation (NORAD, Grant No. RAF-3058 KEN-18/0005); and the European Commission (Grant No. DCI-FOOD/2018/402-634).<br><br>We also acknowledge the International Centre of Insect Physiology and Ecology (icipe) core support provided by the Foreign, Commonwealth and Development Office (FCDO), UK; the Swedish International Development Cooperation Agency (Sida); the Swiss Agency for Development and Cooperation (SDC); Germany's Federal Ministry for Economic Cooperation and Development (BMZ); the Federal Democratic Republic of Ethiopia; and the Kenyan Government.

and **will appear in the published article** if the submission is accepted. Please make sure it is accurate.<br><br>**Unfunded studies**<br>Enter: *The author(s) received no specific funding for this work.*<br><br>**Funded studies**<br>Enter a statement with the following details:<br>• Initials of the authors who received each award<br>• Grant numbers awarded to each author<br>• The full name of each funder<br>• URL of each funder website<br>• Did the sponsors or funders play any role in the study design, data collection and analysis, decision to publish, or preparation of the manuscript?<br>• **NO** - Include this sentence at the end of your statement: *The funders had no role in study design, data collection and analysis, decision to publish, or preparation of the manuscript.*<br>• **YES** - Specify the role(s) played.<br><br>* typeset	The funders had no role in study design, data collection and analysis, decision to publish, or preparation of the manuscript.
**Competing Interests**<br><br>Use the instructions below to enter a competing interest statement for this submission. On behalf of all authors, disclose any competing interests that could be perceived to bias this work—acknowledging all financial support and any other relevant financial or non-financial competing interests.<br><br>This statement is required for submission and **will appear in the published article** if the submission is accepted. Please make sure it is accurate and that any funding sources listed in your Funding Information later in the submission form are also declared in your Financial Disclosure statement.<br><br>View published research articles from *PLOS ONE* for specific examples.	The authors have declared that no competing interests exist.

**NO authors have competing interests**<br><br>Enter: *The authors have declared that no competing interests exist.*<br><br>**Authors with competing interests**<br><br>Enter competing interest details beginning with this statement:<br><br>*I have read the journal's policy and the authors of this manuscript have the following competing interests: [insert competing interests here]*<br><br>* typeset	
**Ethics Statement**<br><br>Enter an ethics statement for this submission. This statement is required if the study involved:<br><br>• Human participants<br>• Human specimens or tissue<br>• Vertebrate animals or cephalopods<br>• Vertebrate embryos or tissues<br>• Field research<br><br>Write "N/A" if the submission does not require an ethics statement.<br><br>General guidance is provided below. Consult the submission guidelines for detailed instructions. **Make sure that all information entered here is included in the Methods section of the manuscript.**	For the community survey that the authors have collected the questionnaire had an introductory statement that sought the respondent's consent to participate in the survey. The icipe science committee has also reviewed and approved the study.

Powered by Editorial Manager® and ProduXion Manager® from Aries Systems Corporation

**Format for specific study types**<br><br>**Human Subject Research (involving human participants and/or tissue)**<br>• Give the name of the institutional review board or ethics committee that approved the study<br>• Include the approval number and/or a statement indicating approval of this research<br>• Indicate the form of consent obtained (written/oral) or the reason that consent was not obtained (e.g. the data were analyzed anonymously)<br><br>**Animal Research (involving vertebrate animals, embryos or tissues)**<br>• Provide the name of the Institutional Animal Care and Use Committee (IACUC) or other relevant ethics board that reviewed the study protocol, and indicate whether they approved this research or granted a formal waiver of ethical approval<br>• Include an approval number if one was obtained<br>• If the study involved *non-human primates*, add *additional details* about animal welfare and steps taken to ameliorate suffering<br>• If anesthesia, euthanasia, or any kind of animal sacrifice is part of the study, include briefly which substances and/or methods were applied<br><br>**Field Research**<br><br>Include the following details if this study involves the collection of plant, animal, or other materials from a natural setting:<br>• Field permit number<br>• Name of the institution or relevant body that granted permission	
**Data Availability**<br><br>Authors are required to make all data underlying the findings described fully available, without restriction, and from the time of publication. PLOS allows rare exceptions to address legal and ethical concerns. See the PLOS Data Policy and FAQ for detailed information.	Yes - all data are fully available without restriction

A Data Availability Statement describing where the data can be found is required at submission. Your answers to this question constitute the Data Availability Statement and **will be published in the article**, if accepted.<br><br>**Important:** Stating 'data available on request from the author' is not sufficient. If your data are only available upon request, select 'No' for the first question and explain your exceptional situation in the text box.<br><br>Do the authors confirm that all data underlying the findings described in their manuscript are fully available without restriction?	
**Describe where the data may be found in full sentences. If you are copying our sample text, replace any instances of XXX with the appropriate details.**<br><br>• If the data are **held or will be held in a public repository**, include URLs, accession numbers or DOIs. If this information will only be available after acceptance, indicate this by ticking the box below. For example: *All XXX files are available from the XXX database (accession number(s) XXX, XXX.).*<br>• If the data are all contained **within the manuscript and/or Supporting Information files**, enter the following: *All relevant data are within the manuscript and its Supporting Information files.*<br>• If neither of these applies but you are able to provide **details of access elsewhere**, with or without limitations, please do so. For example:<br><br>*Data cannot be shared publicly because of [XXX]. Data are available from the XXX Institutional Data Access / Ethics Committee (contact via XXX) for researchers who meet the criteria for access to confidential data.*<br><br>*The data underlying the results presented in the study are available from (include the name of the third party*	The community survey data will be available after acceptance. The household survey are available upon request of the Central Statistics Agency of Ethiopia (https://www.statsethiopia.gov.et/).

and contact information or URL).<br>• This text is appropriate if the data are owned by a third party and authors do not have permission to share the data.<br><br>* typeset	
Additional data availability information:	Tick here if the URLs/accession numbers/DOIs will be available only after acceptance of the manuscript for publication so that we can ensure their inclusion before publication.

Powered by Editorial Manager® and ProduXion Manager® from Aries Systems Corporation

Manuscript

Click here to access/download;Manuscript;Fall armyworm_Impact.docx

The socioeconomic and health impacts of Fall Armyworm in Ethiopia

Zewdu Abro [a], Emily Kimathi [b], Hugo De Groote [c], Tadele Tefera [a], Subramanian Sevgan [b], Saliou Niassy [b], and Menale Kassie [b*]

[a] International Centre of Insect Physiology and Ecology (*icipe*), Addis Ababa, Ethiopia

[b] International Centre of Insect Physiology and Ecology (*icipe*), Nairobi, Kenya

[c] International Maize and Wheat Improvement Center (CIMMYT), Nairobi, Kenya

[*] Corresponding author: mkasse@icipe.org

Abstract

Since 2016, fall armyworm (FAW) has threatened the fragile food systems and economic performance of sub-Saharan Africa. Yet, there is limited evidence on this transboundary pest's economic and food security impacts in the region. Additionally, the health and environmental consequences of the insecticides used to control FAW have not been studied. In this paper, we present evidence on the impacts of FAW on maize production, food security, and human and environmental health. We use a combination of an agroecology-based community survey and nationally representative data from an agricultural household survey to achieve our objectives. The results indicate that the pest causes an average annual loss of 36% in maize production, a reduction of 0.67 million tonnes of maize (0.225 million tonnes per year) between 2017 and 2019. The total economic loss is US $ 200 million, or 0.08% of the gross domestic product. The lost production could have met the per capita maize consumption of 4 million people. We also find that the application of insecticides to control FAW has more significant

toxic effects on the environment than on humans. This paper highlights governments and development partners need to invest in more diverse FAW control strategies that reduce production losses while protecting the environment and biodiversity.

Keywords: Fall armyworm, maize production loss, food security, health, community and household surveys, insecticides

1. Introduction

Maize is a staple food for more than 300 million Africans [1,2]. Despite the importance, its production is constrained by several biotic and abiotic factors that contribute to sub-Saharan Africa's (SSA) pervasive food insecurity. For a long time, stemborers and Striga weed were the main maize pests in SSA, a combination known to cause complete maize production failure [3]. The recent invasion (since 2016) of maize by the fall armyworm (FAW), *Spodoptera frugiperda,* hereafter referred to as FAW, has exacerbated the already fragile food systems and food security in the region [4–8]. Farm-level estimates in some SSA countries show that FAW causes maize production losses of between 11% and 67% [6,9–14].

Infestation by invasive transboundary pests such as FAW also causes additional costs due to insecticide use and labor to control the pest [10,15–17]. The application of insecticides is the primary FAW control strategy in SSA countries [18]. This inevitably has impacts beyond abating maize production losses; insecticides pollution can have adverse effects on the environment, biodiversity, and health of the producers and consumers [12,19–22]. Furthermore, FAW invasions can affect trade, income, and food consumption due to reductions in maize supply. FAW invasions can also increase health expenditure arising from exposure to insecticides and affect the performance of businesses along the maize value chain, such as maize input suppliers and contributors to the livestock feed sector [23–25]. Unless effective control strategies are implemented, the pest will continue to cause massive destruction to maize and affect the livelihoods of millions of people in SSA. Implementing such control strategies requires updates on the current impact of FAW on the economy, food security, and health (human and environmental).

Despite FAW's importance, there are limited studies on its impact on production, the cost of control, including the cost of insecticides, and the unintended negative consequences of insecticide use on human and environmental health. Using survey data from Ghana and Zambia, Day et al. (2017) and Rwomushana et al. (2018) extrapolated production losses due to FAW for twelve SSA countries. Country-specific studies are crucial because the effects of FAW vary across and within countries due to differences in agro-ecology and farm and farmer characteristics. De Groote et al. (2020) show that losses caused by the FAW vary by agro-ecological zones. Many of the existing studies do not capture the large degree of agro-ecological and socioeconomic heterogeneity of smallholder farmers in SSA because the studies rely on limited geographical areas [9,10,13,26]. Many of these studies also use data collected at the early stages of the FAW invasion. The real impacts of FAW infestation may take time to become evident as the infestation varies from season to season. The arrival of the FAW has changed the dynamic of existing farming system constraints to maize production, leading to a new status quo [27].

Invasion by FAW has significantly increased insecticide use in most invaded regions [10,17]. The majority of studies have focused on the efficacy of insecticide for the management of FAW in the invaded regions [28,29], but the increased use of insecticides for FAW control is affecting the health of farmers. For instance, in Ghana and Zambia, farmers have reported sickness after applying insecticides recommended for controlling FAW [12]. However, no systematic study to document the health and environmental effects of insecticides.

In this paper, we present evidence on the economic and health cost of FAW. Particularly, we estimate the pest's effect on maize production, food security, and the effect of insecticide use on public and environmental health. The evidence will help to prioritize

investment in FAW management strategies that simultaneously reduce losses and maintain ecological balance. As secondary objectives, we endeavor to understand farmers' current FAW control measures and the effectiveness and the support that communities receive in combating the pest. Since the accuracy of production loss estimates depends on farmers' knowledge of the pest [30], we examine farmers' awareness of and knowledge about FAW.

To measure FAW's effect on maize production and achieve the secondary objectives, we combined agroecology-based community surveys with nationally representative datasets collected by Ethiopia's Central Statistical Agency (CSA). We covered 150 villages/communities and 1,100 farmers distributed across 30 districts of maize-growing agro-ecological zones. We also collected data from 180 agricultural experts in these communities to validate the results. The community and expert opinion survey data helped us triangulate yield losses to avoid over/underestimation because we recorded community-level yield losses validated through focus group discussions (FGDs). This also reduced data collection costs and saved time as we did not need to interview farmers individually [6]. The datasets from the CSA's Agricultural Sample Survey show a good picture of maize production in Ethiopia. On average, the data covers 17,833 maize-growing farmers across the country. We developed a simple arithmetic formula to quantify maize production losses at the national level. Using secondary data obtained from the Ministry of Agriculture, we applied the environmental impact quotient (EIQ) approach to quantify adverse health risks and the environmental effects of insecticides used to manage FAW [31,32].

We report three key results. First, 97% and 88% of the farmers interviewed were aware of and identified FAW, respectively. Knowledge of farmer's awareness of FAW is vital to estimating its effects accurately. Second, FAW has a considerable socioeconomic impact in

Ethiopia that varies by agro-ecology. From 2017 to 2019, the country lost 0.67 million tonnes of maize production, worth US$ 200 million (0.08% of the Gross Domestic Product). This lost maize could have met the maize consumption requirement of 4 million food-insecure households. Third, FAW has a negative spillover effect on biodiversity and the human population. In the short term, the application of insecticides to control FAW has greater toxic effects on the environment than on humans. However, it can aggravate food insecurity by killing beneficial insects and contaminating other essential natural resources in the long term.

Overall, our findings present a cautionary note about the impacts of FAW. Lack of appropriate control measures against FAW combined with other production constraints can lead to high economic losses to society and monetary expenditures associated with managing this pest. Although the food security cost is not high at the household level (60 kg per year per affected farmer), the economic and biodiversity losses are high at the national level. For example, from 2017 and 2019, the country lost US $ 204 million worth of income due to maize production losses and chemical purchases. However, if the pest persists, it can cause food security and poverty problems in the long run by reducing marketed surplus and income [10].

The rest of the article is structured as follows. In Section 2, we outline study areas and data sources. In Section 3, we describe the estimation approaches used to measure production losses due to FAW and the impact of insecticides on human and environmental health. We present and discuss the results in Section 4. Finally, we make concluding remarks in Section 5.

2. Study areas and data sources

2.1. Study areas

In Ethiopia, FAW is a threat to over 9 million maize-growing farm households. It was first observed in 2017 [33] and is currently one of the most destructive maize pests. Maize is an economically important and strategic food security crop covering 20% of cultivated land and accounting for 30% of the total cereal production [34]. It provides the largest share of calories (22%) for most Ethiopians [35]. It is also the most productive cereal crop in the country, with an average yield of 4 tonnes/ha [34].

This study covers the major maize-producing districts and agro-ecological zones of Ethiopia. We used the sampling frame prepared by the Sustainable Intensification of Maize-Legume Cropping Systems in Eastern and Southern Africa (SIMLESA) project of the International Wheat and Maize Improvement Center (CIMMYT) [36]. The SIMLESA survey was designed to represent the key maize-producing agro-ecological zones of Ethiopia. It covered 225 maize-producing villages in 39 districts in Amhara, Benishangul Gumuz, Oromia, Southern Nations and Nationalities (SNNP), and the Tigray Regional States. In our study, we cover 30 districts and 150 villages. We dropped seven districts in the Oromia Regional State due to security reasons and excluded the Benishangul Gumuz and the Tigray Regional States for logistical reasons. The three remaining Regional States (Amhara, Oromia, and SNNPR) jointly produce more than 86% of the country's maize, as reported in Table 1 [34].

[Table 1 here]

The study villages and their corresponding agro-ecological zones, represented by the CIMMYT's maize mega-environments (MMEs), are represented in Figure 1. The MMEs are homogenous production environments defined based on agro-climatic conditions [37,38] and classified using maximum rainfall and temperature. Rainfall and temperature are key parameters that affect maize production and the biology and spread of the FAW [39,40].

[Figure 1 here]

Figure 1. The study areas and the location of sample communities within maize mega-environments

Among the communities we surveyed, 71 are classified as wet upper mid-altitudes, 48 are in the highlands, 28 fall in the dry mid-altitudes, two are found in the wet lower mid-altitudes, and one is in the dry lowlands. Over the study period, nearly 96% of the maize production in the country came from three major MMEs: the wet upper mid-altitudes (45%), the highlands (39%), and the dry mid-altitudes (12%). The remainder of the country's maize production came from the wet lower mid-altitudes, wet lowlands, and dry lowlands, each contributing nearly 1% (~~see~~ Table 1).

2.2. Data sources and collection

We used data from three sources: first, we used primary community and expert opinion survey datasets collected for this study using FGDs between June and July 2020 from 150 communities. On average, seven farmers participated per FGD, making 1100 farmers (10%

women) in total. The expert opinion survey involved 180 agricultural experts, of whom 150 were development agents who worked with the farmers, and 30 were experts who worked in the districts' agriculture offices. We used a structured questionnaire covering various topics, including farmers' awareness and knowledge of FAW, the percentage of farmers affected by FAW, control strategies, attainable yield, actual yield, and yield losses due to FAW. We collected data on the percentage of farmers affected by FAW in their respective villages and the yield losses due to FAW in 2017, 2018, and 2019 production seasons. To understand farmers' levels of FAW awareness and knowledge, we asked each FGD participant two questions: (1) Are you aware of the FAW? and (2) Can you identify the FAW from these pictures? (~~See~~ Figure 2). The questionnaire had an introductory statement that sought the respondent's consent to participate in the survey. The *icipe* science committee has also reviewed and approved the study.

Second, we used the agricultural sample survey datasets for 2017, 2018, and 2019 main seasons. These datasets are nationally representative household survey data collected by the Ethiopian Central Statistical Agency [34]. The agency was obtained verbal consent from respondents.

Third, we used insecticide data collected by the authors from the Ministry of Agriculture [41].

[Figure 2 here]

Figure 2. Pictures of lepidopterous insect pests shown to farmers: A) stemborers (either Chilo partellus (A1), or Busseola Fusca (A2); B) African armyworm; and C) fall armyworm

To measure the effect of FAW, we combined the community survey data with the CSA data, from which we obtained the total maize area and the number of maize-growing farmers in the country CSA datasets. We identified the agro-ecological zones for each survey community by overlaying their coordinates with the global maize mega-environments’ shapefile. Because we did not have access to the farmers’ coordinates in the CSA survey, we used the centroids of the CSA’s survey areas to identify the key MMEs. Finally, we used region, zone, district, and MMEs as unique identifiers to combine the two datasets.

3. Estimation approach

3.1. Measuring maize production losses

Maize yield loss is the difference between attainable yield without the presence of FAW and actual yield in the presence of FAW [6]. However, FAW is not the only cause of yield loss. Several other factors contribute to yield loss, including abiotic factors (e.g., drought and soil fertility) and other biotic factors (e.g., diseases, stemborers, and locusts). Results may be biased if farmers are asked directly to estimate yield loss due to FAW alone without considering the potential yield loss attributable to other production constraints. To mitigate this problem, we first asked farmers to compute the actual maize yield in the community in the presence of all production constraints, including FAW. Secondly, we asked them to estimate the attainable yield in the absence of production constraints. Thirdly, we asked farmers to

quantify FAW's contribution and other production constraints to the yield gap, which is the difference between the attainable and actual yields.

We calculated the total production loss (PL_i) due to FAW using equation (1) as follows:

$$PL_i = \sum_i^k A_i \times [(Y_a - Y) \times L_i] \times (N_{hi} \times F_{ai}) \quad (1)$$

where the index i represents agro-ecological zones; k denotes the number of agro-ecological zones; A_i is the average land size (ha) devoted to maize in that zone; Y_a is the attainable yield without the presence of production stresses, including FAW (tonnes/ha); and Y is the actual yield in the presence of FAW and other production stresses (tonnes/ha). L_i is the proportion of the average yield losses attributed to FAW (%); N_h is the number of maize-growing households; and F_{ai} is the proportion of farmers affected by FAW. We obtained the values for Y_a, Y, L_i and F_{ai} from the community survey data, while the values of A_i and N_{hi} were from the CSA datasets (Table 2).

[Table 2 here]

Although farmers and government incur management costs, we focused on production losses (PL_i) because we did not have full management cost data. However, we report the chemical costs and measure the impacts of chemical spraying on human health and the environment, as discussed in the next section.

3.2. Measuring the impacts of insecticides on environmental and human health

The sudden invasion of FAW has forced the government and stakeholders to use insecticides as an emergency measure in FAW-affected maize fields. Over the study period, the government distributed 457,427 liters of insecticides, sprayed on 1.5 million ha of maize

(see Table 8). The direct cost of pesticides to the government was about US $4 million. This does not include the insecticides that farmers purchased themselves or the costs of surveillance and management, on which no data are available.

While insecticides are used to boost crop productivity, they have unintended consequences on human and environmental health. The use of insecticides poses a risk to human health, water quality, food safety, aquatic species, and beneficial insects [42–48]. To measure the risks to human health and the environment caused by pesticides used to control FAW, we used the environmental impact quotient (EIQ) [31,32]. The EIQ has three components: producer, consumer, and environmental effects. The producers' and consumers' effects measure the potential health impact of direct exposure to insecticides and food and water contaminated with insecticides. Insecticides like chlorpyrifos have been shown to harm children's cognitive development, while others have been linked to cancer [49]. The environmental effects of insecticides include the threat on fish, birds, bees, and other beneficial insects and potential leaching. Although the EIQ uses arbitrary weights to measure the effects of the insecticides, it has been used in other studies as there is no readily available alternative to EIQ at present [21,45,50,51]. In any event, it is vital to consider the health and environmental effects [21]. For the detailed computation of EIQ, we refer readers to Kovach et al. (1992) and Grant (2020).

4. Results and discussion

4.1. Farmers awareness of and knowledge in identifying FAW

We found that 97% of the FGD participants were aware of FAW. Moreover, 88% of the farmers in the FGDs correctly identified the FAW ~~from the pictures shown~~ (Table 3), slightly

more than those in Kenya (82%) (De Groote et al., 2020). There were relatively fewer farmers in the wet upper mid-altitude and highland MMEs that correctly identified FAW than farmers in other agro-ecological zones. These two agro-ecological zones contribute more than 80% of the country's maize production, suggesting that the extension system may need additional capacity-building activities for farmers.

[Table 3 here]

4.2. Farmers' FAW control strategies

It seems the effectiveness of FAW control strategies varied by agro-ecology (Figure 3). Farmers in the wet lower mid-latitude and dry lowland agro-ecological zones named a few control methods. We asked farmers to score their effectiveness on a scale from zero (minimum) to ten (maximum). Insecticides received an average score of six (Figure 3 Panel A). The effectiveness of chemicals remained the highest (Figure 3, Panels B-F). Cultural (e.g., rotation and fallow) and biological (e.g., caring for the striped earwig species during field management) pest control techniques received a score of five. The effectiveness of botanical extracts (e.g., neems) and mechanical control (e.g., killing larvae of the pest) received below-average scores. The FGD participants gave a low effectiveness score (two) for agro-ecological approaches (e.g., cropping systems such as intercropping), which are promoted to control FAW in Ethiopia and elsewhere [2,7,18,52]. This result is in line with Kassie et al. (2020), who found that intercropping (maize-legume) had little impact on controlling maize production losses due to FAW in southern Ethiopia. However, an experimental study in Uganda showed that intercropping was more effective than monocropping in controlling FAW and stemborers

[53]. A systematic study of intercropping, differentiated by country and agro-ecology, may determine its effectiveness.

[Figure 3 here]

Figure 3. Farmers' FAW control strategies by agro-ecological zones

4.3. External support for FAW control

The impact of the pest may depend on the type of support the community receives to manage the pest. We asked the FGD participants to assess if the community they belonged to had received external support for controlling FAW. We also asked whether the support had increased, decreased, or remained the same. The support included training in FAW management, provision of credit, free insecticides, and spraying equipment. Farmers receive support from the regional and federal governments, Agricultural Research Systems, and development organizations. More than half of the communities (61%) had not received any support (Table 4). For 6% of the communities, support had remained the same, suggesting that farmers had received continuous support since the first occurrence of FAW in their respective communities. About 21% of the communities reported that external support for FAW control had increased. On the other hand, 11% of the studied communities reported that their communities had received external support, but it had decreased over time. The absence or low level of support may have contributed to higher production losses.

[Table 4 here]

4.4. Farmers affected by Fall Armyworm

The map in Figure 4 shows the distribution of the affected farmers by agro-ecology. The FGD results indicated that FAW affected 40% of maize farmers (Table 5), while the expert opinion interviews estimated that 51% of the farmers were affected (Table A1, Appendix). In the wet lower mid-altitudes, which contain 3% of all maize farmers (Table A2, Appendix), farmers were the most affected (59%). In the dry lowlands, where 4% of maize farmers are located, FAW affected 17% of them. For the other agro-ecological zones, the proportion of farmers affected by FAW was close to the country's average at 40%. The total number of farmers affected by FAW over the study period was 3.7 million per annum.

[Table 5 here]

[Figure 4 here]

Figure 4. Geographic distribution of average farmers affected (%) by FAW, 2017-2019

4.5. Maize yield losses due to FAW

We estimated that, on average, FAW causes an annual loss of 36% in maize production (Table 6), despite the use of control measures. This estimate was close to the agricultural experts' estimate of 32% (Table A1, Appendix). The map in Figure 5 shows the distribution of the yield losses by agro-ecology. In the wet mid-altitudes and highland agro-ecological zones, losses were close to the country's average of 36%. However, yield losses in the dry lowlands were higher than the country's average. This was perhaps because of the limited support

farmers had received for FAW control in this zone (Table 4), the resulting limited use of control strategies (Figure 3), and the absence of hosts other than maize. The variability in yield loss could be due to several factors, including farming practices, natural enemies' availability, and climatic factors [18]. Several studies have established the role of climatic factors in FAW incidence. The combined effect of natural enemies, including predators and parasitoids, could be up to 60% effective in controlling FAW if these natural enemies were conserved [28,54]. Heavy downpours can reduce FAW by washing away neonates and affecting the flight capability of adult moths. Soil health in terms of soil moisture and fertility enhance plant vigor, which, in turn, protects crops against heavy damage [55,56]. In the future, a detailed study would be warranted to understand the factors driving differences across agro-ecological zones. Our yield loss estimates are lower than those reported by De Groote et al. (2020) in Kenya. However, this comparison should be interpreted with caution as yield losses depend on several factors (agro-ecology, farm management, years of data collection, estimation approach, etc.).

[Table 6 here]

[Figure 5 here]

Figure 5. Geographic distribution of average yield loss (%) due to FAW, 2017-2019

4.6. Production losses: economic and food security implications

This sub-section reports the total maize production losses computed using equation (1), presented by agro-ecological zones (Table 7) and administrative regions (Table A4, Appendix).

In 2017, we estimate that Ethiopia lost 0.18 million tonnes of maize to FAW (Table 7). The production loss increased from 0.22 million tonnes in 2018 to 0.25 million tonnes in 2019. The increase in loss over time could be attributable to changes in the proportion of farmers affected (Table 5), the percentage yield losses (Table 6), the number of maize farmers (Table A2, Appendix), and maize land size (Table A3, Appendix). The highest production losses are in the wet upper mid-altitude, highland, and dry mid-altitude agro-ecological zones. The production losses are small compared to the first estimates by Day et al. (2017) and Rwomushana et al. (2018). For 2017, our estimate was 7% of the 2.74 million tonnes of maize production loss in Ethiopia, estimated by Day et al. (2017). Similarly, our estimated losses were 13% of the 1.67 million tonnes of maize loss in 2018 estimated by Rwomushana et al. (2018).

Over the study period, the total production loss was 0.67 million tonnes of maize, generating an economic loss of US $200 million to the Ethiopian economy (Table 7). The economic loss is equivalent to 0.08% of the country's Gross Domestic Product ($US 262 billion) from 2017 to 2019 [57]. Alternatively, the losses were equivalent to 3% of the total foreign direct investment (US $7,327 million) in 2017 and 2018 alone [4]. Using the 152 kg per capita consumption of maize in Ethiopia [58], the quantity of maize lost could have met the per capita maize consumption of over 50% (4.3 million) of the country's chronically food-insecure (8.5 million people) [59].

Although the economic and food security costs are high at the national level, the food security impact does not seem to be high at the household level-the per capita maize production loss is 60 kg per year (0.22 million tonnes divided by 3.7 million affected framers). Kassie et al. (2020) find no significant effect of FAW on per capita maize consumption at the

household level. However, if the pest persists, it can have food security and poverty implication at the household level by reducing marketed surplus and income [10].

[Table 7 here]

4.7. Human and environmental effects of insecticides used for FAW control

Four insecticides are used in Ethiopia to reduce the impact of the pest (Table 8). According to the World Health Organization (WHO), malathion is slightly hazardous while the rest of the chemicals are moderately hazardous [60]. According to the Leach and Mumford (2008) toxicity-level classification, all these insecticides have a high toxicity impact on the environment (e.g., by killing beneficial insects). Malathion, diazinon, and dimethoate carry considerable risk for the environment, as shown by the high EIQ values (Table 8). Synthetic insecticides are important management options in FAW control, but repeated application increases the accumulation of insecticides in the environment and raises major concern, as demonstrated by the high EIQ values. Furthermore, resistance to major classes of synthetic insecticides in the native regions of this pest is another problem. The efficacy of a synthetic insecticide-based management strategy is not guaranteed, as the FAW has developed resistance to many active ingredients from different classes of insecticides [8,61–63]. This suggests the need for resistance management as a vital component of integrated pest management. The risk impact on human health is relatively low, given the relatively low value of EIQ for consumers and producers. However, repeated exposure to small doses of insecticides can lead to long-term effects in humans. This calls for judicious and appropriate

use of synthetic insecticides to successfully manage FAW and sustain the increased productivity of maize in Ethiopia and elsewhere in Africa. Previous reports show that Ethiopia is home to many natural enemies of the FAW [64]. The adverse impacts of these insecticides on non-target and beneficial organisms and the environment might also explain pest incidence variations and yield losses because of the negative impact of insecticides on biological control agents. Our results suggest the importance of control strategies that effectively suppress the pest without compromising the natural environment. These may include biopesticides [65], predators and parasitoids [64,66], and the push-pull technology [18,67].

[Table 8 here]

5. Conclusions

Data on production losses are crucial for informed management of pests and evaluating the effectiveness of pest control measures. In this paper, we present the first comprehensive estimate of the impact of FAW on maize production, food security, and health in Ethiopia, contributing to the few existing studies in SSA. We used primary community survey data combined with a nationally representative agricultural household survey to achieve our objectives.

The pest caused significant economic and food losses at the country level. About 0.67 million tonnes of maize are lost, equivalent to 2.54% of the maize production (25.96 million tonnes) over the study period. This generates US $200 million (0.08% of the country's GDP) economic loss to the country. These results vary by agro-ecology, which is vital for prioritizing investment. At the current 152 kg per capita consumption of maize in the country, the maize lost to the FAW could have met the maize consumption requirement of over 4 million food-

insecure people. In the long run, together with other co-existing production constraints, FAW can put the livelihoods of many poor people at risk and may reverse the gains already made in productivity and poverty reduction that the country has achieved over the last three decades. In addition to the direct economic and food security losses controlling the pest using pesticides contributes to environmental damage or degradation, thus threatening sustainable food production.

A key implication of these findings is that developing and promoting affordable, accessible, ecologically friendly control strategies must be facilitated to control the pest sustainably. Our analysis does not reflect the total impact of FAW due to limited data. Firstly, we did not capture the full management costs, such as insecticides and labor costs, in controlling the pest. Secondly, although we indicate the toxicity of insecticides for the environment and human health, the chemical application's health and environmental costs (monetary costs to the society) are not factored into the analysis. We recommend future studies to (1) consider both the direct and indirect effects of the pest and its control to reflect its overall cost; and (2) introduce effective, healthy, and environmentally friendly management strategies for FAW and conduct comprehensive evaluations of their effectiveness.

Acknowledgments

We thank Zebdewos Selato and Hulubanchi Abera of the Ministry of Agriculture for their support and for providing data on insecticides for FAW control in Ethiopia. We also thank Solomon Balew for supporting the research by designing the CSPro program during data collection, the enumerators and supervisors for their dedication in conducting the survey, and the farmers and experts who participated in the study. Finally, we thank Rahel Solomon for processing the letters and bringing the datasets from Ethiopia’s Central Statistical Agency.

References

1. Badu-apraku BB, Fakorede MAB, Lum AF. Evaluation of experimental varieties from recurrent selection for striga resistance in two extra-early maize populations in the savannas of West and Central Africa. Exp Agric. 2007;43: 183–200.
2. Matova PM, Kamutando CN, Magorokosho C, Kutywayo D, Gutsa F, Labuschagne M. Fall-armyworm invasion, control practices and resistance breeding in Sub-Saharan Africa. Crop Sci. 2020;60: 2951–2970.
3. De Groote H. Maize yield losses from stemborers in kenya. insect Sci Appl. 2002;22: 89–96.
4. FAO. FAOSTAT. Data. In: Food and Agriculture Organization [Internet]. 2019 [cited 10 Jul 2019]. Available: http://www.fao.org/faostat/en/#data
5. Hruska AJ. Fall armyworm (Spodoptera frugiperda) management by smallholders. CAB Rev Perspect Agric Vet Sci Nutr Nat Resour. 2019;14: 43.
6. De Groote H, Kimenju SC, Munyua B, Palmas S, Kassie M, Bruce A. Spread and impact of fall armyworm (Spodoptera frugiperda J.E. Smith) in maize production areas of Kenya. Agric Ecosyst Environ. 2020;292: 106804.
7. Njuguna E, Nethononda P, Maredia K, Mbabazi R, Kachapulula P, Rowe A, et al. Experiences and perspectives on spodoptera frugiperda (Lepidoptera: Noctuidae) management in Sub-Saharan Africa. J Integr Pest Manag. 2021;12: 1–9.
8. Otim MH, Fiaboe KKM, Akello J, Mudde B, Obonyom AT, Bruce AY, et al. Managing a transboundary pest: The fall armyworm on maize in Africa. 2021. doi:10.5772/intechopen.96637
9. Kumela T, Simiyu J, Sisay B, Likhayo P, Mendesil E, Gohole L, et al. Farmers’

knowledge, perceptions, and management practices of the new invasive pest, fall armyworm (Spodoptera frugiperda) in Ethiopia and Kenya. Int J Pest Manag. 2019;65: 1–9.

10. Kassie M, Wossen T, Groote H De, Tefera T, Sevgan S, Balew S. Economic impacts of fall armyworm and its management strategies: evidence from Southern Ethiopia. Eur Rev Agric Econ. 2020.

11. Day R, Abrahams P, Bateman M, Beale T, Clottey V, Cock M, et al. Fall armyworm: impacts and implications for Africa. Outlooks on pest management. Outlooks Pest Manag. 2017;28: 196–201.

12. Rwomushana I, Bateman M, Beale T, Beseh P, Cameron K, Chiluba M, et al. Fall Armyworm : Impacts and Implications for Africa Evidence Note update, October 2018. Knowledge for Life. CABI. 2018.

13. Baudron F, Zaman-Allah MA, Chaipa I, Chari N, Chinwada P. Understanding the factors influencing fall armyworm (Spodoptera frugiperda J.E. Smith) damage in African smallholder maize fields and quantifying its impact on yield. A case study in Eastern Zimbabwe. Crop Prot. 2019;120: 141–150.

14. Overton K, Maino JL, Day R, Umina PA, Bett B, Carnovale D, et al. Journal Pre-proof Global crop impacts, yield losses and action thresholds for fall armyworm (Spodoptera frugiperda): a review. 2021. doi:10.1016/j.cropro.2021.105641

15. Kansiime MK, Mugambi I, Rwomushana I, Nunda W, Lamontagne-Godwin J, Rware H, et al. Farmer perception of fall armyworm (Spodoptera frugiderda J.E. Smith) and farm-level management practices in Zambia. Pest Manag Sci. 2019;75: 2840–2850.

16. Tambo JA, Day RK, Lamontagne-Godwin J, Silvestri S, Beseh PK, Oppong-Mensah B,

et al. Tackling fall armyworm (Spodoptera frugiperda) outbreak in Africa: an analysis of farmers’ control actions. Int J Pest Manag. 2019;0: 1–13.

17. Yang X, Wyckhuys KAG, Jia X, Nie F, Wu K. Fall armyworm invasion heightens pesticide expenditure among Chinese smallholder farmers. J Environ Manage. 2021;282: 111949.

18. Harrison RD, Thierfelder C, Baudron F, Chinwada P, Midega C, Schaffner U, et al. Agro-ecological options for fall armyworm (Spodoptera frugiperda JE Smith)management: Providing low-cost, smallholder friendly solutions to an invasive pest. J Environ Manage. 2019;243: 318–330.

19. Pingali PL. Environmental consequences of agricultural commercialization in Asia. Environ Dev Econ. 2001;6: 483–502.

20. Lai W. Pesticide use and health outcomes: Evidence from agricultural water pollution in China. J Environ Econ Manage. 2017; In Press.

21. Midingoyi S kifouly G, Kassie M, Muriithi B, Diiro G, Ekesi S. Do Farmers and the Environment Benefit from Adopting Integrated Pest Management Practices? Evidence from Kenya. J Agric Econ. 2019;70: 452–470.

22. Gautam S, Schreinemachers P, Uddin MN, Srinivasan R. Impact of training vegetable farmers in Bangladesh in integrated pest management (IPM). Crop Prot. 2017;102: 161–169.

23. Early R, González-Moreno P, Murphy ST, Day R. Forecasting the global extent of invasion of the cereal pest Spodoptera frugiperda, the fall armyworm. NeoBiota. 2018;50: 25–50.

24. Chapman D, Purse B V., Roy HE, Bullock JM. Global trade networks determine the

distribution of invasive non-native species. Glob Ecol Biogeogr. 2017;26: 907–917.

25. Jeger M, Bragard C, Caffier D, Candresse T, Chatzivassiliou E, Dehnen-Schmutz K, et al. Pest categorisation of Spodoptera frugiperda. EFSA J. 2017;15: 1–32.

26. Koffi D, Kyerematen R, Eziah VY, Osei-Mensah YO, Afreh-Nuamah K, Aboagye E, et al. Assessment of impacts of fall armyworm, Spodoptera frugiperda (Lepidoptera: Noctuidae) on maize production in Ghana. 0.1093/jipm/pmaa015. J Integr Pest Manag. 2020;11.

27. Hailu G, Niassy S, Bässler T, Ochatum N, Studer C, Salifu D, et al. Could fall armyworm, Spodoptera frugiperda (J. E. Smith) invasion in Africa contribute to the displacement of cereal stemborers in maize and sorghum cropping systems. Int J Trop Insect Sci. 2021.

28. Sisay B, Tefera T, Wakgari M, Ayalew G, Mendesil E. The efficacy of selected synthetic insecticides and botanicals against fall armyworm, spodoptera frugiperda, in maize. Insects. 2019;10.

29. Deshmukh S, Pavithra HB, Kalleshwaraswamy CM, Shivanna BK, Maruthi MS, Mota-Sanchez D. Field efficacy of insecticides for management of invasive fall armyworm, Spodoptera frugiperda (J. E. Smith) (Lepidoptera: Noctuidae) on maize in India. Florida Entomol. 2020;103: 221–227.

30. Prasanna BM, Huesing JE, Eddy R, Peschke VM. Fall armyworm in Africa: A guide for integrated pest management, First Edition. Mexico, CDMX: CIMMYT. 2018.

31. Kovach J, Petzoldt C, Degnil J, Tette J. A method to measure the environmental impact of pesticides. New York's Food Life Sci Bull. 1992;139.

32. Grant J. Calculator for Field Use EIQ (Environmental Impact Quotient). New York State Integrated Pest Management Program, Cornell Cooperative Extension, Cornell

University. 2010-2020. 2020.

33. Legesse T. Fall armyworm continues to spread in Ethiopia’s maize fields. Food and Agriculture Organization of the United Nations in Ethiopia. 2017 [cited 14 Oct 2020]. Available: http://www.fao.org/ethiopia/news/detail-events/en/c/1028088/

34. CSA. Agriculture Sample Survey 2018/19 (2011 E.C.) Volume II Report on area and production of major crops (Private Peasant holdings (meher and belg seasons). Volume II & V. Central Statistical Agency. Statistical Bulletin 589. 2019.

35. Dorosh P, Minten B. Ethiopia’s Agrifood System: Past Trends, Present Challenges, and Future Scenarios. Washington, DC: International Food Policy Research Institute. 2020.

36. Jaleta M, Kassie M, Marenya P, Yirga C, Ernstein O. Impact of Improved Maize Variety Adoption on Household Food Security in Ethiopia: An Endogenous Switching Regression Approach. Food Secur. 2018;10: 81–93.

37. Sonder K. Global map of maize mega-environments. https://hdl.handle.net/11529/10624, CIMMYT Research Data & Software Repository Network, V4. (2016). 2016.

38. Bellon MR, Hodson D, Bergvinson D, Beck D, Martinez-Romero E, Montoya Y. Targeting agricultural research to benefit poor farmers: Relating poverty mapping to maize environments in Mexico. Food Policy. 2005;30: 476–492.

39. Ramirez-Cabral NYZ, Kumar L, Shabani F. Future climate scenarios project a decrease in the risk of fall armyworm outbreaks. J Agric Sci. 2017;155: 1219–1238.

40. Kasoma C, Shimelis H, Laing MD. Fall armyworm invasion in Africa: implications for maize production and breeding. J Crop Improv. 2020;00: 1–36.

41. MoA. Pesticides used for fall armyworm control in Ethiopia (2017/18-2019/20). Raw datasets. Ministry of Agriculture, Ethiopia. 2020.

42. Athukorala W, Wilson C, Robinson T. Determinants of health costs due to farmers' exposure to pesticides: An empirical analysis. J Agric Econ. 2012;63: 158–174.

43. Arias-Estévez M, López-Periago E, Martínez-Carballo E, Simal-Gándara, J. Mejuto JC, García-Río L. The mobility and degradation of pesticides in soils and the pollution of groundwater resources. Agriculture, Ecosystems and Environment, 123, 247–260. Agric Ecosyst Environ. 2008;123: 247–260.

44. Liu H, Cheng, Wang XH. A general study on Chinese diet: Pesticide residue. J Hyg Res. 1995;24: 356–360.

45. Kouser S, Qaim M. Bt Cotton , Pesticide Use and Environmental Efficiency in Pakistan. J Agric Econ. 2015;66: 66–86.

46. Mullen JD, Norton GW, Reaves DW. Economic analysis of environmental benefits of integrated pest management. Econ J Agric Appl Econ. 1997;29: 243–254.

47. Skevas T, Stefanou SE, Lansink AO. Do farmers internalise environmental spillovers of pesticides in production? J Agric Econ. 2013;64: 624–640.

48. Leach AW, Mumford JD. Pesticide Environmental Accounting: A method for assessing the external costs of individual pesticide applications. Environ Pollut. 2008;151: 139–147.

49. Liu J, Schelar E. Pesticide Exposure and Child Neurodevelopment Summary and Implications. Work Heal Saftey. 2012.

50. Sharma R, Peshin R. Impact of integrated pest management of vegetables on pesticide use in subtropical Jammu, India. Crop Prot. 2016;84: 105–112.

51. Kniss A, Coburn C. Quantitative evaluation of the environmental impact quotient (EIQ) for comparing herbicides. PLoS One. 2015;10: e0131200.

52. Salato Z, Crozier J. Fall armyworm on maize. Spodoptera frugiperda. “Ye amerika mete temch”. Pest management decision guide: Green and Yellow List. Plantwise. Ministry of Agriculture and CABI. https://www.cabi.org/isc/FullTextPDF/2017/20177800723.pdf. 2017.

53. Hailu G, Niassy S, Zeyaur KR, Ochatum N, Subramanian S. Maize–legume intercropping and push–pull for management of fall armyworm, stemborers, and striga in Uganda. Agron J. 2018;110: 2513–2522.

54. Sisay B, Simiyu J, Malusi P, Likhayo P, Mendesil E, Elibariki N, et al. First report of the fall armyworm, Spodoptera frugiperda (Lepidoptera: Noctuidae), natural enemies from Africa. J Econ Entomol. 2018;142: 800–804.

55. Baltzer JL, Davies SJ, Jennifer Baltzer C. Rainfall seasonality and pest pressure as determinants of tropical tree species’ distributions. Ecol Evol. 2012;2: 2682–2694.

56. Wyckhuys KAG, Oõneil RJ. Local agro-ecological knowledge and its relationship to farmersÕ pest management decision making in rural Honduras. Agric Human Values. 2007;24: 307–321.

57. World Bank. World Development Indicators. The World Bank. 2020.

58. Muricho G, Kassie M, Marenya P, Yirga C, Tostao E, Mishili F, et al. Identifying socioeconomic constraints to and incentives for faster technology adoption: pathways to sustainable intensification in eastern and southern Africa. Cross Country Report for Adoption Pathways 2013 Surveys (adoption pathways). 2014.

59. IPC. Ethiopia: Belg pastoral and agropastoral producing areas analysis. The Integrated Food Security Phase Classification (IPC) Acute Food Insecurity Analysis. http://www.ipcinfo.org/fileadmin/user_upload/ipcinfo/docs/IPC%20Ethiopia%20AcuteFoo

dSec%202020July20. 2020.

60. WHO. The WHO recommended classification of pesticides by hazard and guidelines to classification 2009. World Health Organization. 2009.

61. Gutiérrez-Moreno R, Mota-Sanchez D, Blanco CA, Whalon ME, Terán-Santofimio H, Rodriguez-Maciel JC, et al. Field-evolved resistance of the fall armyworm (Lepidoptera: Noctuidae) to synthetic insecticides in Puerto Rico and Mexico. J Econ Entomol. 2019;112: 792–802.

62. Özkara A, Akyıl D, Konuk M. Pesticides, Environmental Pollution, and Health. In: Larramendy ML, Soloneski S, editors. Environmental Health Risk - Hazardous Factors to Living Species. 2016.

63. Yu SJ. Insecticide resistance in the fall armyworm, Spodoptera frugiperda (J. E. Smith). Pestic Biochem Physiol. 1991;39: 84–91.

64. Sisay B, Simiyu J, Mendesil E, Likhayo P, Ayalew G, Mohamed S, et al. Fall armyworm, spodoptera frugiperda, infestations in East Africa: Assessment of damage and parasitism. Insects. 2019;10: 195–205.

65. Akutse KS, Kimemia JW, Ekesi S, Khamis FM, Ombura OL, Subramanian S. Ovicidal effects of entomopathogenic fungal isolates on the invasive Fall armyworm Spodoptera frugiperda (Lepidoptera: Noctuidae). J Appl Entomol. 2019;143: 626–634.

66. Laminou SA, Ba MN, Karimoune L, Doumma A, Muniappan R. Parasitism of locally recruited egg parasitoids of the fall armyworm in Africa. Insects. 2020;11: 430–443.

67. Midega CAO, Pittchar JO, Pickett JA, Hailu GW, Khan ZRA. Climate-adapted push-pullsystem effectively controls fall armyworm, Spodoptera frugiperda (J E Smith), in maize in EastAfrica. Crop Prot. 2018;105: 10–15.

Tables

Table 1. Area under maize cultivation and production by agro-ecological zones

	Cultivated land (millions of ha)			Production (millions of tonnes)		
Agro-ecological zones	2017	2018	2019	2017	2018	2019
Wet upper mid-altitudes	0.85	0.97	0.85	3.74	4.27	3.78
Wet lower mid-altitudes	0.05	0.03	0.03	0.14	0.09	0.09
Dry mid-altitudes	0.34	0.29	0.34	1.00	0.88	1.37
Wet lowlands	0.01	0.03	0.03	0.04	0.11	0.13
Dry lowlands	0.04	0.04	0.02	0.08	0.05	0.07
Highlands	0.69	0.85	0.82	2.95	3.59	3.59
Total	1.98	2.20	2.08	7.95	8.98	9.03

Source: CSA's agricultural sample survey

Table 2. Attainable yield, actual yield, and average land size, 2017-2019

Agro-ecological zones	Attainable yield (tonnes/ha)- (Y_a)	Actual yield (tonnes/ha)- (Y)	Yield losses due to FAW and other stresses (tonnes/ha)- ($Y_a - Y$)	Average land size (ha)- (A_i)	Number of farmers (millions) (N_{hi})
	A	B	C=A-B	D	E
Wet upper mid-altitudes	4.02	2.76	1.26	0.12	3.41
	(0.10)	(0.08)	(0.05)	(0.08)	(0.015)
Wet lower mid-altitudes	5.08	3.73	1.35	0.08	0.32
	(0.90)	(0.82)	(0.15)	(0.01)	(0.006)
Dry mid-altitudes	4.40	2.86	1.54	0.14	1.29
	(0.13)	(0.13)	(0.07)	(0.01)	(0.013)
Dry lowlands	3.10	2.47	0.63	0.10	0.33
	(0.12)	(0.16)	(0.11)	(0.02)	(0.007)
Highlands	4.13	2.88	1.25	0.11	3.80
	(0.14)	(0.11)	(0.06)	(0.06)	(0.014)
Average	4.11	2.82	1.29	0.12	9.28
	(0.07)	(0.06)	(0.03)	(0.04)	(0.007)

Note: Standard errors in parenthesis.

Sources: columns A and B are from the community survey data; columns D and E are from the CSA's agricultural sample survey.

Table 3. Farmers awareness and knowledge of FAW (%)

Agro-ecological zones	Awareness of FAW (%)	Correctly identified FAW (%)
Wet upper mid-altitudes	92	70
Wet lower mid-altitudes	100	100
Dry mid-altitudes	99	88
Dry lowlands	100	100
Highlands	92	80
Average	97	88

Source: Community survey

Table 4. FAW control support to communities

External support:	Wet Upper Mid-altitudes	Wet Lower Mid-altitudes	Dry Mid-altitudes	Dry Lowlands	Highlan
Not at all	61	100	63	0	61
Increased	21	0	30	0	18
Same	7	0	0	100	5
Decreased	10	0	5	0	15
Do not know	2	0	1	0	0
Total	100	100	100	100	100

Source: Community survey

Table 5. Proportion of farmers affected by FAW (%)

Agro-ecological zones	2017	2018	2019	Average
Wet upper mid-altitudes	36.40	39.64	39.71	38.60
	(2.51)	(2.49)	(2.28)	(1.40)
Wet lower mid-altitudes	55.00	55.00	67.50	59.17
	(20.00)	(25.00)	(27.50)	(11.21)
Dry mid-altitudes	44.02	41.71	45.27	43.68
	(4.73)	(5.07)	(4.96)	(2.82)
Dry lowlands	20.00	12.50	17.50	16.67
	(0.00) [a]	(2.50)	(2.50)	(1.67)
Highlands	37.95	42.39	44.75	41.67
	(3.58)	(4.06)	(4.07)	(2.25)
Average	38.07	40.68	42.13	40.30
	(1.86)	(1.97)	(1.89)	(1.10)

Note: Standard errors of the mean are reported in parenthesis; [a] the standard errors are zero because FGD participants provided 20% loss for all data points

Source: Community survey

Table 6. Yield losses due to FAW (%)

Agro-ecological zones	2017	2018	2019	Average
Wet upper mid-altitudes	34.37	34.71	35.82	34.97
	(2.14)	(1.96)	(2.05)	(1.18)
Wet lower mid-altitudes	35.00	32.50	35.00	34.17
	(5.00)	(2.50)	(5.00)	(2.01)
Dry mid-altitudes	38.78	41.21	43.46	41.17
	(3.78)	(3.47)	(3.15)	(1.99)
Dry lowlands	80.00	80.00	80.00	80.00
	(0.00) [a]	(0.00) [a]	(0.00) [a]	(0.00) [a]
Highlands	33.20	36.43	34.20	34.61
	(3.13)	(2.69)	(2.75)	(1.65)
Average	35.13	36.64	36.96	36.25
	(1.61)	(1.45)	(1.48)	(0.87)

Note: Standard errors in parenthesis; [a] the standard errors are zero because FGD participants provided 80% loss for all data points

Source: Community survey

Table 7. Estimated total maize production losses

	Loss (millions of tonnes)			Loss (millions of $US) ¥		
MMEs	2017	2018	2019	2017	2018	2019
Wet upper mid-altitudes	0.064	0.080	0.084	15.21	22.35	28.53
Wet lower mid-altitudes	0.007	0.004	0.006	1.52	0.89	1.73
Dry mid-altitudes	0.049	0.047	0.073	13.33	14.94	24.87
Wet lowlands	0.002	0.005	0.005	0.55	1.37	1.53
Dry lowlands	0.002	0.002	0.002	0.75	0.58	0.46
Highlands	0.057	0.089	0.095	14.22	25.24	32.28
Total	0.182	0.228	0.265	45.59	65.38	89.40

¥ We use producer prices to estimate the value of production losses. The exchange rate was 26.87 ETB/$US in 2017, 27.43 ETB/$US in 2018, and 29.23 ETB/$US in 2019.

Source: authors' computation based on community survey and CSA's agricultural sample survey

Table 8. Human health and environmental impacts of insecticides use to control FAW

				Components of field use EIQ			
Insecticides	Active ingredient (%)	Application rate (liter/ha)	Quantity (liters)	Average EIQ	Consumer effects	Producer effects	Ecological effects
Malathion	50	2	114,529	23.80	3.80	7.70	49.60
Diazinon	60	1	256,914	22.60	1.30	3.50	63.00
Dimethoate	40	1	25,488	11.50	3.90	3.50	26.90
Chlorpyrifos	48	0.5	60,496	5.50	0.40	1.20	14.90

Source: authors' computation based on MoA's pesticides data (MoA, 2020)

Appendix

Table A1. Estimates of farmers affected and yield losses reported by agricultural experts (2017-2019)

Maize mega-environments	Farmers affected (%)	Yield loss (%)
Wet upper mid-altitudes	55	32
Wet lower mid-altitudes	79	40
Dry mid-altitudes	44	32
Dry lowlands	67	43
Highlands	47	30
Average	51	32

Source: experts' survey (2017-2019)

Table A2. Number of farmers producing maize

	Number of farmers (millions)		
Agro-ecological zones	2017	2018	2019
Wet upper mid-altitudes	3.28	3.33	3.62
Wet lower mid-altitudes	0.43	0.29	0.24
Dry mid-altitudes	1.24	1.11	1.51
Wet lowlands	0.10	0.16	0.13
Dry lowlands	0.41	0.30	0.29
Highlands	3.28	3.69	4.41
Total	8.75	8.87	10.20

Source: CSA's agricultural sample survey.

Table A3. Average land size per household

Agro-ecological zones	2017	2018	2019
Wet upper mid-altitudes	0.11	0.13	0.12
Wet lower mid-altitudes	0.06	0.08	0.09
Dry mid-altitudes	0.14	0.14	0.15
Wet lowlands	0.09	0.21	0.21
Dry lowlands	0.11	0.11	0.08
Highlands	0.10	0.11	0.12
Overall average	0.11	0.12	0.12

Source: CSA’s agricultural sample survey.

Table A4. Zone and regional-level estimates of maize production losses

		Loss (tonnes)		
Region	Zone	2017	2018	2019
Amhara	Semen Gondar	7,036	808	775
Amhara	Debub Gondar	5,698	8,267	12,989
Amhara	Semen Wollo	1,566	1,003	1,387
Amhara	Debub Wollo	1,024	2,963	2,827
Amhara	Semen Shewa	1,650	1,545	936
Amhara	Misrak Gojjam	8,777	9,244	11,778
Amhara	Mirab Gojjam	7,456	14,868	18,693
Amhara	Waghimra	1,835	715	485
Amhara	Awi	4,667	8,412	5,068
Amhara	Oromia Liyu Zone	1,764	235	504
Amhara	Bahir Dar Liyu		1,675	339
Amhara	Argoba Liyu	774	95	
Amhara	Dessie Town Administration		481	60
Amhara	Gondar Ketema Liyu Zone		408	265
Amhara	Maekelawi Gondar		10,785	8,548
Amhara	Mirab Gondar		1,648	1,710
	Amhara	42,245	63,150	66,364
Oromia	Mirab Wollega	6,652	5,470	5,360
Oromia	Misrak Wollega	6,338	7,544	5,964
Oromia	Ilu Ababor	10,688	3,721	4,947

		Loss (tonnes)		
Region	Zone	2017	2018	2019
Oromia	Jimma	4,278	11,470	11,230
Oromia	Mirab Shewa	3,784	4,555	6,356
Oromia	Semen Shewa	576	718	758
Oromia	Misrak Shewa	7,545	11,804	21,663
Oromia	Arsi	3,728	6,087	7,629
Oromia	Mirab Hararghe	1,751	4,356	3,369
Oromia	Misrak Hararghe	1,074	3,275	2,817
Oromia	Bale	4,613	4,044	4,652
Oromia	Borena	2,696	54	418
Oromia	Debub Mirab Shewa	3,522	2,421	3,251
Oromia	Guji	5,320	2,651	5,643
Oromia	Mirab Guji		1,130	1,713
Oromia	Oromia Liyu Zone		413	209
Oromia	Mirab Arsi	6,373	10,323	14,363
Oromia	Kelem Wollega	4,986	4,187	3,752
Oromia	Horo Guduru Wollega	9,859	8,064	4,531
Oromia	Buno Bedele		3,473	5,249
	Oromia	83,783	95,759	113,874
SNNP	Gurage	3,072	4,281	5,074
SNNP	Hadiya	2,889	4,303	3,925
SNNP	Kembata Tembaro	1,356	1,350	954

		Loss (tonnes)		
Region	Zone	2017	2018	2019
SNNP	Sidama	1,248	2,211	5,844
SNNP	Gedeo	878	417	1,667
SNNP	Welayta	1,857	2,052	5,229
SNNP	Debub Omo	5,535	5,562	7,389
SNNP	Sheka	159	302	1,170
SNNP	Kefa	3,079	5,709	6,868
SNNP	Gamo Gofa	5,093	9,758	11,145
SNNP	Bench Maji	4,550	5,592	3,484
SNNP	Yem Liyu	2,027	1,279	622
SNNP	Segen Akababi Hizboch	9,058	6,883	5,572
SNNP	Alaba Special	5,235	3,999	3,184
SNNP	Dawro	1,203	954	1,864
SNNP	Basketo Special	2,728	623	302
SNNP	Konta Liyu	640	243	265
SNNP	Silite	5,565	7,987	8,857
	SNNP	56,171	63,505	73,414
	Ethiopia ¥	182,199	222,414	253,652

¥ Note that the grand total is slightly different from the total loss reported in Table 7. This is due to differences in the calculation of averages by maize mega-environments and zones.

Sources: authors’ estimate based on community survey and CSA’s agricultural sample survey.

Supporting information

S1 File. Data processing approach: stata do file.

S2 File. The communty survey data: Stata Dataset file.

Manuscript

Click here to access/download;Figure;Fig 1.tif

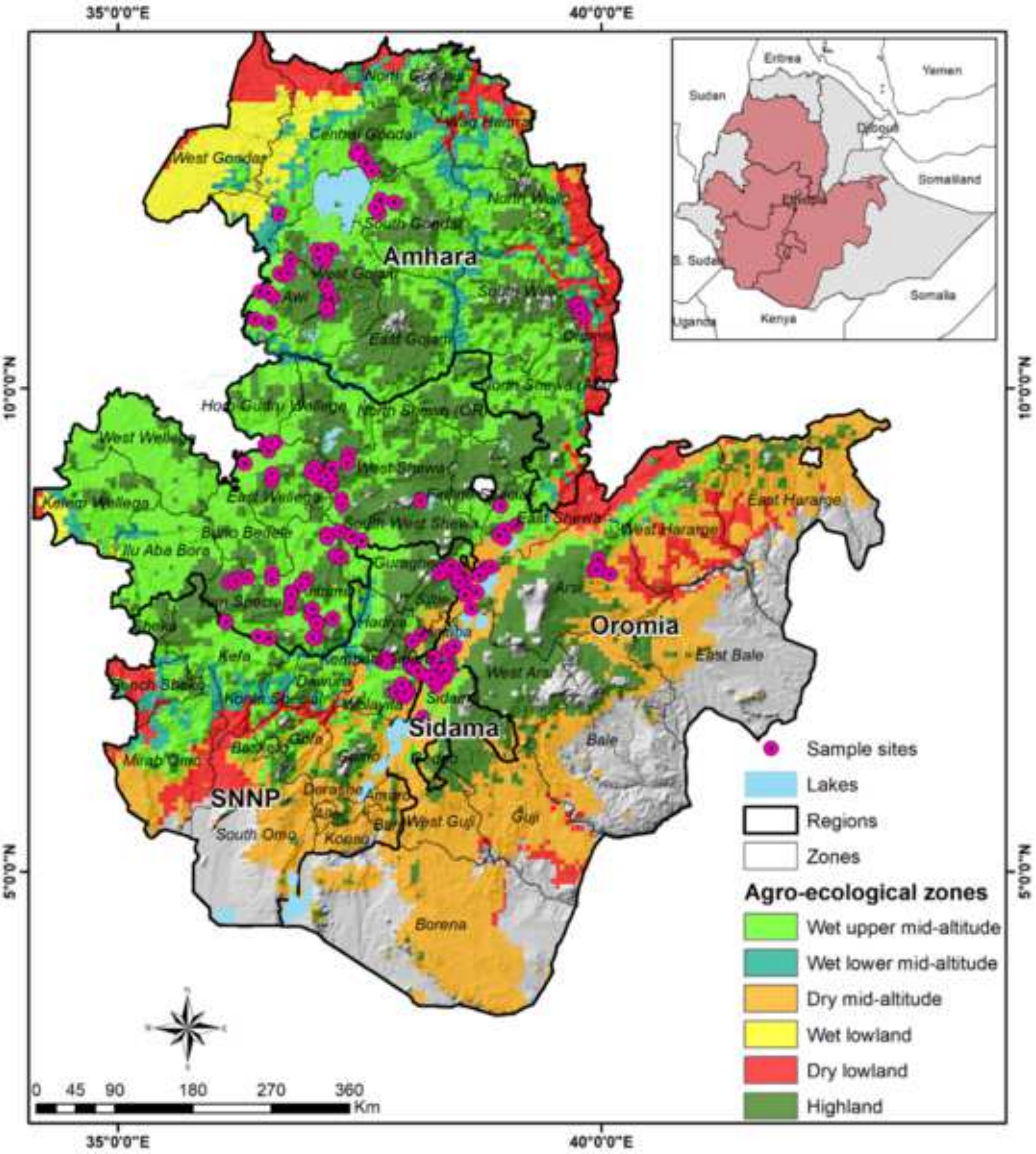

Figure Click here to access/download;Figure;Fig 2.tif

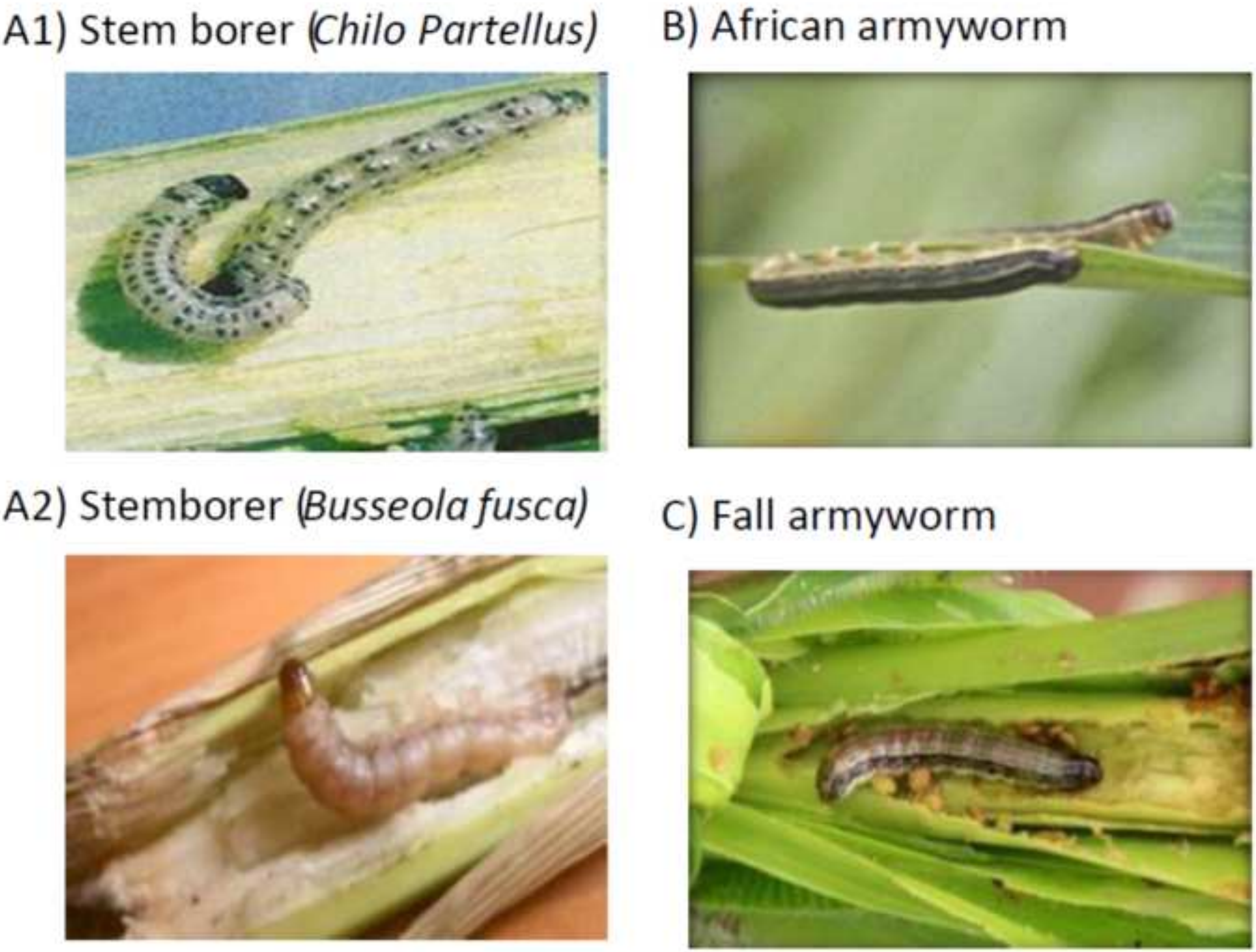

Figure

Click here to access/download;Figure;Fig 3.tif

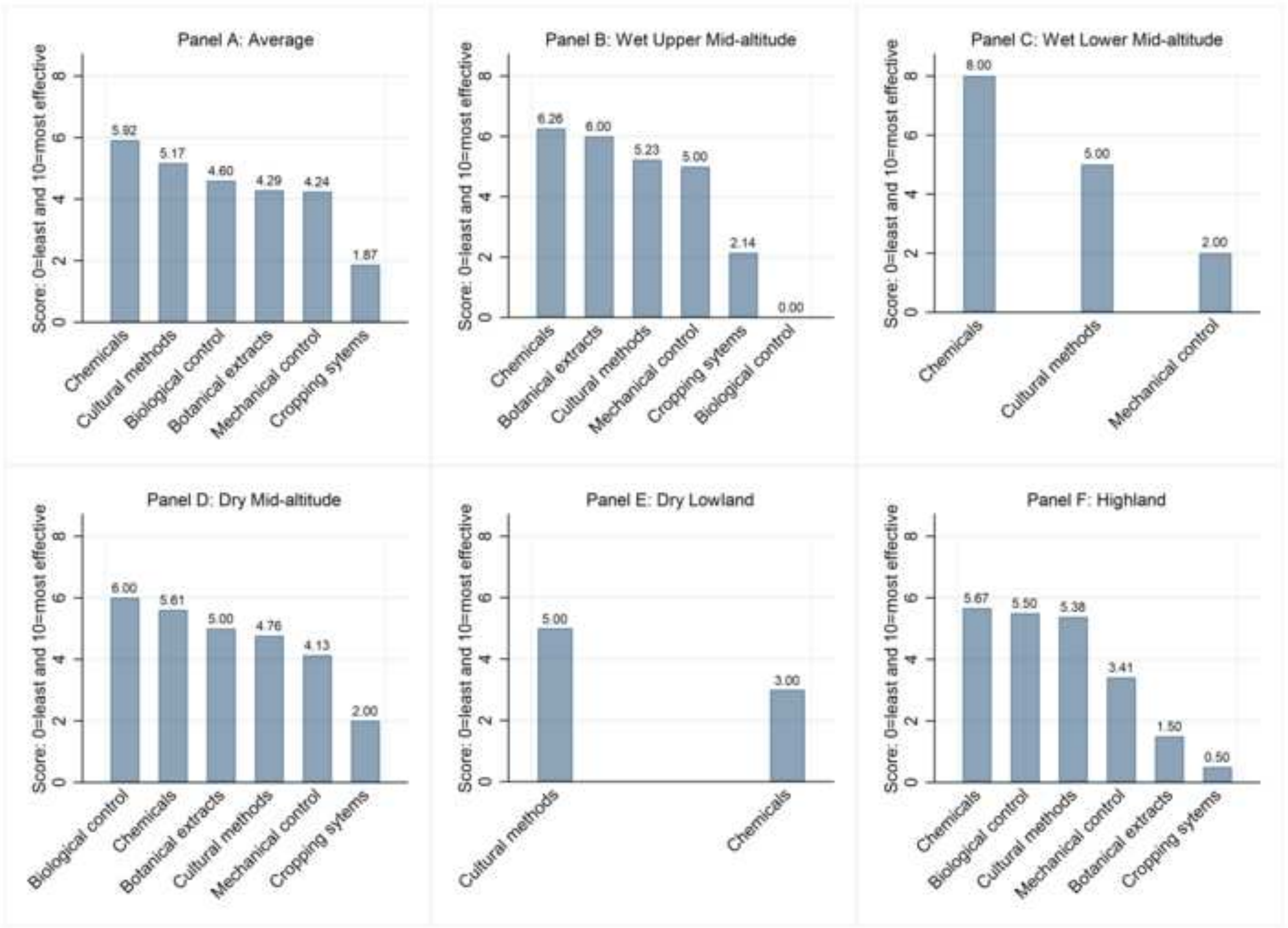

Figure
Click here to access/download;Figure;Fig 4.tif

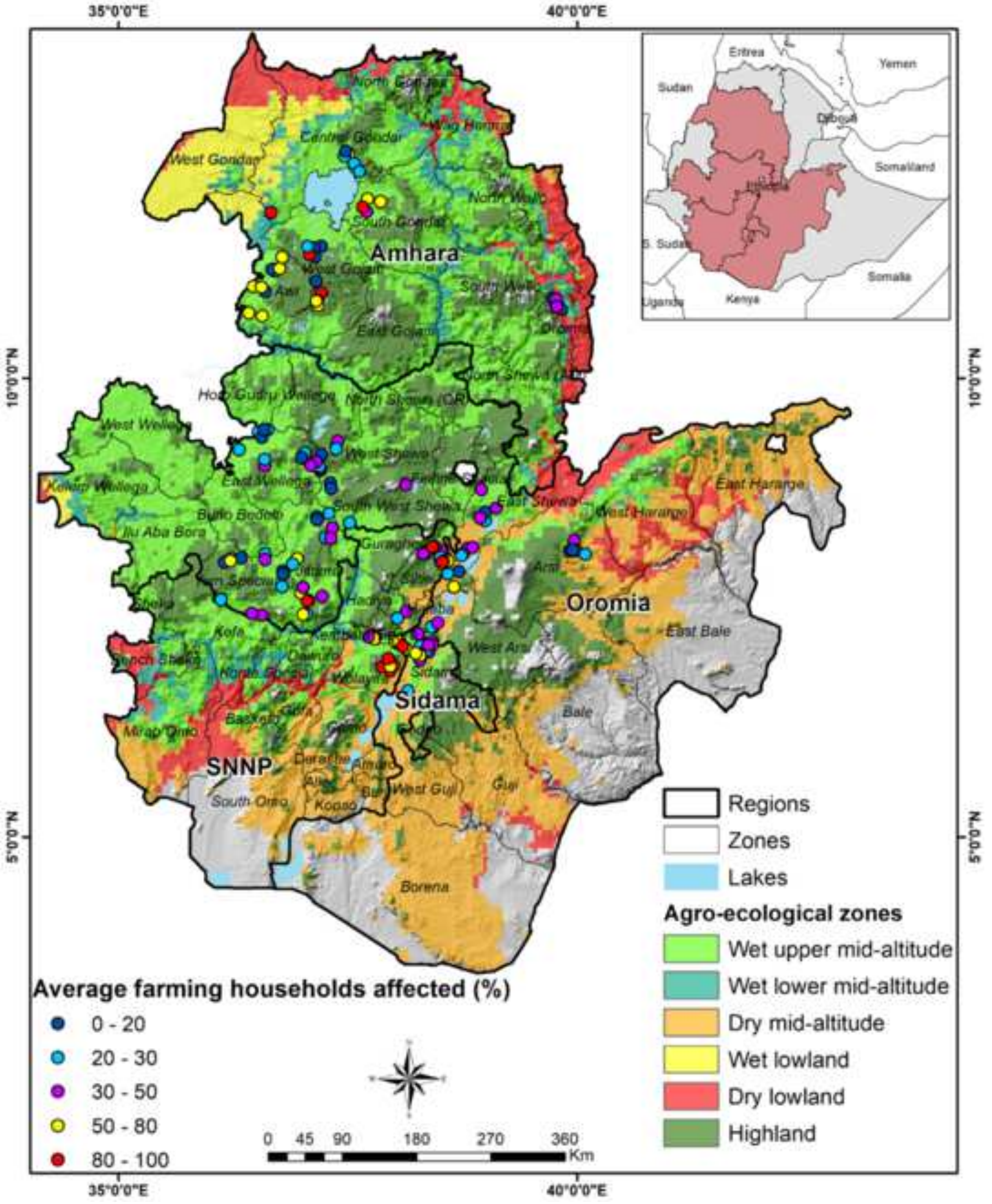

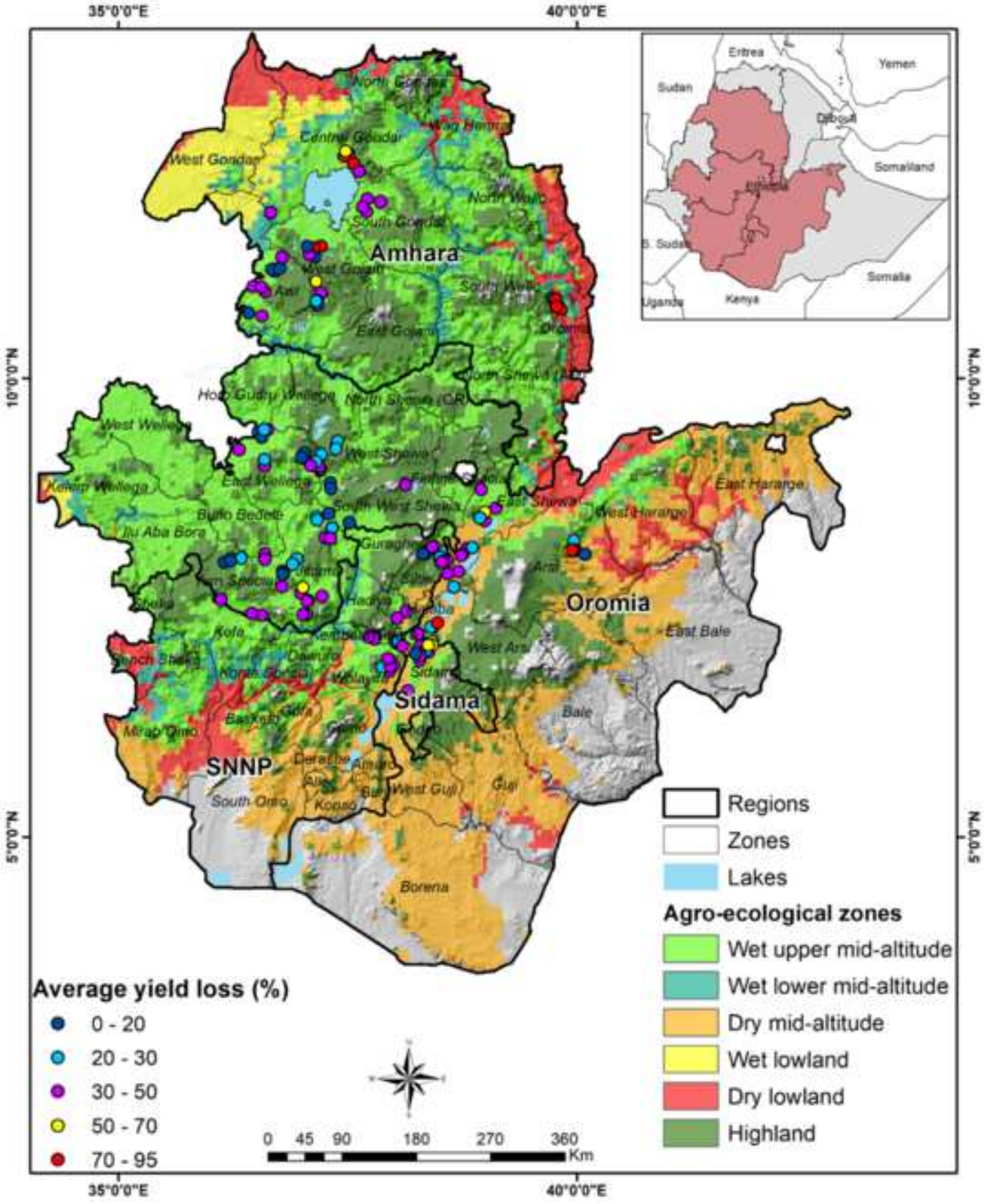
35°0'0"E
40°0'0"E
10°0'0"N
5°0'0"N
Eritrea
Yemen
Sudan
Djibouti
Somaliland
Ethiopia
S. Sudan
Somalia
Uganda
Kenya
Amhara
Oromia
Sidama
SNNP
Central Gondar
West Gondar
North Gondar
South Gondar
West Gojam
East Gojam
Awi
North Wollo
South Wollo
North Shewa (OR)
Horo Guduru Wellega
West Wellega
Kelem Wellega
East Wellega
West Shewa
South West Shewa
East Shewa
West Hararge
East Hararge
Buno Bedele
Ilu Aba Bora
Jimma
Guraghe
Hadiya
Arsi
West Arsi
East Bale
Bale
Sidama
Kefa
Sheka
Bench Sheka
Dawuro
Wolayita
Gofa
Basketo
Mirab Omo
Derashe
Amaro
Alle
Burji
Konso
West Guji
Guji
South Omo
Borena
Regions
Zones
Lakes
Agro-ecological zones
Wet upper mid-altitude
Wet lower mid-altitude
Dry mid-altitude
Wet lowland
Dry lowland
Highland
Average yield loss (%)
0 - 20
20 - 30
30 - 50
50 - 70
70 - 95
0
45
90
180
270
360
Km

Click here to access/download
Supporting Information
do_file_socioeconomic_and_health_impacts_of_Fall
Armyworm.pdf

Click here to access/download
Supporting Information - Compressed/ZIP File Archive
Community_survey_dataset.rar
