# Supplementary material for: Socioeconomic and health impacts of fall armyworm in Ethiopia
Source: PLoS One. 2021 Nov 4;16(11):e0257736. doi: 10.1371/journal.pone.0257736 (PMC8568106; doi:10.1371/journal.pone.0257736)
Supplement: Attachment — Submitted filename: Responses_to_Reviewer#2_feedback.docx [file pone.0257736.s005.docx]

**Responses to reviewer # 2 feedback**

We thank the reviewer for the helpful comments, which have greatly improved our article. We have incorporated your suggestions into the revised manuscript. Please see below our point-by-point responses to your specific feedback.

| S.N. | Reviewer’s feedback | Authors’ responses |
| --- | --- | --- |
| 1. | 1. It is questionable if the FGD participants were able to remember the actual and attainable yields in 2017 and 2018 when the FGDs were conducted in June-July 2020. Moreover, in lines 202-204, the authors wrote “Secondly, we asked them to estimate the attainable yield in the absence of production constraints. Thirdly, we asked farmers to quantify FAW’s contribution and other production constraints to the yield gap….”. Are you really sure that the farmers could reliably indicate how much yield they would have obtained in the absence of production constraints and the contribution of FAW? Thus, the yield loss estimates of 36% or 0.67 million tonnes of maize and the total economic loss to Ethiopia are based on data that are likely to be fraught with recall bias and measurement errors, raising questions about their reliability. The authors need to reflect on these concerns. I would have thought that a more reliable method would have been to compare the yield estimates of FAW-affected and non-affected households, as was done by a previous related study in Ethiopia, i.e., Kassie et al. 2020. | Thank you. You raised a very valid point. Indeed, recall bias is there even scientific methods to measure yield are prone to errors (Kosmowski et al.,2021; Abay et al.,2019) and need to be acknowledged. We have acknowledged this in the revised version ( line numbers 178-190 and 446-448 of the cleaned version of the paper).  Although we cannot entirely out recall bias, we developed confidence in farmers’ estimates on yields and losses for the following reasons.  (1) We recorded maize yield and yield loss agreed by each member of the FGD, often after a hot debate. This can help to minimize recall bias and avoid too high or too low estimates. A recent study (Abay et al., 2019) in Ethiopia shows that difference in self-reported and objectively measured yield is not big. Even in some cases, farmer’s reported yield is better than yield objectively measured by W-walk and Transect methods (Kosmowski et al.,2021).  (2) Farmers cultivate maize on the same plot year after year, and production variation across years is limited-implying recalling recent years' data might not be difficult. We have checked this using our recent household panel data in the southern region of Ethiopia. The first wave data was used by Kassie et al. The coefficient of variation is 056 and 0,54 for the first and second wave data, respectively.  (3)Because of the community-level campaigns and intensive information exchange through extension and other local communication channels to control FAW, farmers provided strong attention to the pest and its impact, which can help them provide reasonable estimates of yields and losses. We also took the average estimates from various farmers groups in each agroecological zone, which may cancel out noises and errors at the farmer and village levels.  (4) Crop production is farmers’ jobs and livelihood. We believe that farmers know agricultural production well and can estimate and project what their fields can provide. They also have a good understanding of the relative importance of various production constraints, including FAW. This enables them to estimate losses due to various production constraints.    (5) The data were collected after well-trained enumerators and supervisors carefully explained until the FGD participants understand the questions  (5) On average, the estimated values we obtained are stable across the three study years, indicating that the farmers' estimates based on recall do not deviate much across years, so the impact of estimate across year may not significantly influence the potential impact of FAW.  Yes, the approach used by Kassie et al. is indeed another option. However, Kassie et al.’s paper based on a regression model reported the average effects for the entire sample. The coefficient is the average value like the FGD for all sample households and applies to the community as a whole of that particular study area. In principle, in the FGD, we take the average value of sample households (group members). Kassie et al.’s approach also needs collecting plot-level data across various farming systems and agroecological zones throughout the country with a large sample size in each of them, which was time-consuming and expensive. It also needs estimating regressions to each village to obtain yield losses in each respective village.  References  Abay, K.A., Abate, G.T., Barrett, C.B., Bernard, T.,  2019. Correlated non-classical measurement errors,  ‘Second best’ policy inference, and the inverse size  productivity relationship in agriculture. Journal of  Development Economics 139, 171-184.  Kosmowski, F., Chamberlin ., J., Ayalew, H., Sida, T.,  Abya, K., and Carufurd, P. How accurate are yield  estimates from crop cuts? Evidence from smallholder  maize farms in Ethiopia. Food Policy. 2021;102"1-10. |
| 2. | 2. The authors found that while 97% of the FGD participants were aware of FAW, only 88% were able to correctly identify FAW. One may wonder how a farmer can be aware of FAW when they cannot identify it? In this context, it would be helpful to explain what is considered to be awareness of FAW. Does awareness imply that a farmer has at least heard about FAW? | We have indicated the awareness and knowledge are about FAW (see line numbers 193-196 of the cleaned version of the paper). Awareness implies the farmers have heard about the pest through peer-to-peer information exchange or another information channel such as radio. Farmers can be aware of the pest, but they may not necessarily know the pest unless they experienced it or took practical training about it. Knowledge is much more than awareness, and farmers must identify FAW from the pictures to say they are knowledgeable of the pest. |
| 3. | 3. In lines 248-250, the authors stated “Moreover, 88% of the 249 farmers in the FGDs correctly identified the FAW from the pictures shown (Table 3), slightly more than those in Kenya (82%) (De Groote et al., 2020).” Out of curiosity, I went to look at the De Groote et al. (2020) paper and found that the four pictures of insect pests labelled A1, A2, B and C in your manuscript are exactly as those presented in the De Groote et al. (2020) paper. Surprisingly, you did not attribute the source of the pictures to De Groote et al. (2020) or indicate the original source, if any. Wouldn’t this give rise to an issue of copyright infringement? | Thank you for pointing this out. We agree that respecting copyright is important for us, and not attributing the source was wrong. The De Groote et al. (2020) paper where we took the picture from was cited in the earlier versions of the manuscript but mistakenly deleted in the submitted version. We are sorry for this, and we have now cited the paper in the revised version of the manuscript. Two of the coauthors of De Groote et al. (2020) are also coauthors of this manuscript, and they have provided their consent. Moreover, De Groote et al. (2020) is open access, which allows us to reuse it. Additionally, the Agriculture, Ecosystems, and Environment said their consent is not needed as the paper was open access. |
| 4. | 4. In lines 297-298, the authors wrote “…while the expert opinion interviews estimated that 51% of the farmers were affected (Table A1, Appendix)”. This percentage and others in Table A1 seem to be unreliable and based on expert guesstimates because there is no indication that the 180 experts sought the opinions of farmers on FAW or observed farmers’ fields for FAW attack. How did the experts know how many farmers were affected by FAW? Same applies to the expert estimates of yield loss due to FAW. | The experts we considered are the development agents (DAs) and their district-level supervisors. About 83% of the 180 experts were development agents, and the remaining 17% were their district-level supervisors. The DAs closely work with farmers, conduct scouting exercises to monitor and manage pests, and distribute insecticides to control the pest. They are responsible for reporting production- and related data to the district agricultural office, including areas affected by FAW and numbers of farmers affected in their respective command areas. Therefore, their estimate can be judged reliable though may not be as reliable as farmers’ yield loss data.  We have the above information in the revised version (See lines 162-170, cleaned version of the paper). |
| 5. | 5. Finally, I have reservations that you were actually able to estimate the effect of FAW on human health and the environment based on the data used. For example, it is indicated in the abstract that “We also find that the application of insecticides to control FAW has more significant toxic effects on the environment than on humans”. However, you did not provide any evidence on insecticides used by the sampled farmers for FAW control. Your EIQ estimations were based on MoA’s pesticides data (government distributed pesticides) and application rates that may not reflect the farmers’ actual pesticide application practices against FAW. Moreover, the use of these pesticides may not have an effect on human health if the farmers use the appropriate personal protective equipment and observe the necessary safe pesticide practices, and vice versa. It appears your results may rather be reflective of the potential health and environmental effects of the listed pesticides in Ethiopia, regardless of whether or not they are used for FAW control. | Thank you very much for these important points. We measure the direct cost of FAWt at the country level, regardless of its effect on individual framers. We follow the same approach for measuring the effects of already used insecticides to control FAW on health and the environment, i.e., measuring its impact at the country level. In sum, the main objective of measuring the health and environmental impacts of insecticides used to control FAW was to account for the indirect cost of the pest, regardless of who used the insecticides and their effects on individual framers (see line numbers 250-252 of the cleaned version of the paper). We measure the actual, not the potential impact, as the insecticides were already used in farmers' fields. Framer level data helps to understand the distributional effects of insecticides, but this is not our objective.  The Ministry of Agriculture (MoA) distributed insecticides to districts affected by the pest through its regional agricultural offices. We have collected the names of the insecticides used by farmers from the development agents and district-level supervisors. Although we did not ask farmers, all the insecticides distributed by the MoA were mentioned by the DAs.  You are right; insecticides might not impact health if farmers use personal protective equipment and observe safety measures. However, our experience and evidence in developing countries show farmers rarely use proper safety measures (e.g., Ghimire and Woodward, 2013; Rwomushana et al., 2018; see line numbers 252-254 of the cleaned version of the paper).  References  1)Ghimire, N. and Woodward, R. T. ‘Under- and over-use of pesticides: An international analysis’, Ecological Economics, Vol. 89, (2013) pp. 73–81  2)Rwomushana, I., Bateman, M., Beale, T., Beseh, P., Cameron, K., Chiluba, M., Clottey, V., Davis, T., Day, R., Early, R., Godwin, J., Gonzalez-Moreno, P., Kansiime, M., Kenis, M., Makale, F., Mugambi, I., Murphy, S., W., N., Phiri, N., Pratt, C., Tambo, J., 2018. Fall Armyworm : Impacts and Implications for Africa Evidence Note update, October 2018. Knowledge for Life. CABI. |
